# A systematic review of methods to estimate colorectal cancer incidence using population-based cancer registries

**DOI:** 10.1186/s12874-022-01632-7

**Published:** 2022-05-19

**Authors:** Norah Alsadhan, Alaa Almaiman, Mar Pujades-Rodriguez, Cathy Brennan, Farag Shuweihdi, Sultana A. Alhurishi, Robert M. West

**Affiliations:** 1 Department of Community Health Sciences, College of Applied Medical Sciences, King Saud University, Riyadh, Kingdom of Saudi Arabia; 2 School of Medicine, Leeds Institute of Health Sciences, University of Leeds, Leeds, UK

**Keywords:** Incidence, Colorectal cancer, Cancer registry, Methods, Reporting, Population-based study, Systematic review

## Abstract

**Background:**

Epidemiological studies of incidence play an essential role in quantifying disease burden, resource planning, and informing public health policies. A variety of measures for estimating cancer incidence have been used. Appropriate reporting of incidence calculations is essential to enable clear interpretation. This review uses colorectal cancer (CRC) as an exemplar to summarize and describe variation in commonly employed incidence measures and evaluate the quality of reporting incidence methods.

**Methods:**

We searched four databases for CRC incidence studies published between January 2010 and May 2020. Two independent reviewers screened all titles and abstracts. Eligible studies were population-based cancer registry studies evaluating CRC incidence. We extracted data on study characteristics and author-defined criteria for assessing the quality of reporting incidence. We used descriptive statistics to summarize the information.

**Results:**

This review retrieved 165 relevant articles. The age-standardized incidence rate (ASR) (80%) was the most commonly reported incidence measure, and the 2000 U.S. standard population the most commonly used reference population (39%). Slightly more than half (54%) of the studies reported CRC incidence stratified by anatomical site. The quality of reporting incidence methods was suboptimal. Of all included studies: 45 (27%) failed to report the classification system used to define CRC; 63 (38%) did not report CRC codes; and only 20 (12%) documented excluding certain CRC cases from the numerator. Concerning the denominator estimation: 61% of studies failed to state the source of population data; 24 (15%) indicated census years; 10 (6%) reported the method used to estimate yearly population counts; and only 5 (3%) explicitly explained the population size estimation procedure to calculate the overall average incidence rate. Thirty-three (20%) studies reported the confidence interval for incidence, and only 7 (4%) documented methods for dealing with missing data.

**Conclusion:**

This review identified variations in incidence calculation and inadequate reporting of methods. We outlined recommendations to optimize incidence estimation and reporting practices. There is a need to establish clear guidelines for incidence reporting to facilitate assessment of the validity and interpretation of reported incidence.

**Supplementary Information:**

The online version contains supplementary material available at 10.1186/s12874-022-01632-7.

## Introduction

Epidemiological studies of incidence play an essential role in quantifying disease burden, healthcare resource planning, and informing public health policies. Incidence is a crucial measure of epidemiology representing the number of new disease cases in a specific population divided by the population’s size at risk during a particular period [1]. A variety of measures for estimating cancer incidence in population-based studies have been reported in the literature. The magnitude and interpretation of incidence estimates depend on methodological choices such as the definition of numerator and denominator and the standard population used to calculate the age-standardized rate (ASR) [1–4].

Variations in incidence calculation influence comparisons of regional and global rates and trends and their interpretation. Thus, crucial requirements for generating comparable and reproducible incidence statistics include: i) a precise definition of the disease of interest with a specification of the classification used and coding, ideally validated within data source; ii) a clear description of the numerator data and the population at risk; and iii) an explicit explanation of the methods used to estimate denominator size [1, 5, 6]. Additionally, quantifying and reporting uncertainty around health estimates in population-based studies is imperative to inform readers who draw conclusions from these estimates [7, 8].

Population-based studies often utilize data from cancer registries to derive incidence statistics. The primary purpose of these registries is to provide a reliable source of information for assessing cancer risk. The International Agency for Research on Cancer (IARC) advises registries to continually evaluate data quality by several quantitative and qualitative methods [5, 9]. Yet, the extent of detail provided by researchers about these quality indicators remains unclear, and results of evaluations are rarely publicly available.

Furthermore, cancer registries rely on trained registrars to abstract data from patients’ medical records. Some abstracted data may be incomplete due to human error or poor quality documentation within the medical record, leading to inaccurate and missing values within cancer registries [10]. Thus, quantification of missingness, explicit and detailed reporting of assumptions and handling of missing data help readers make informed interpretations of the findings.

There is a growing body of evidence to suggest that the level of reproducibility in scientific research is inadequate. Poor reporting of incidence methods might negatively affect research findings’ credibility, comparability, and reproducibility [11]. The Enhancing the Quality and Transparency of Health Research (EQUATOR) network provides reporting guidelines for observational studies; yet none of the current guidelines adequately address the reporting of methods used in measuring incidence.

Because it was not practical to consider all cancers in this study, we chose colorectal cancer (CRC) as an exemplar. CRC is of particular interest due to its increasing global burden among women and men and to the role of screening in prevention and early detection. CRC is a type of cancer that starts in the rectum or colon. CRC can be categorized into three sub-types based on its anatomical site: proximal colon, distal colon, and rectum [12]. According to the GLOBOCAN 2020 estimates of cancer incidence, CRC is the third most common cancer and the second leading cause of cancer-related deaths worldwide [13]. The rate of CRC has been steadily increasing in some regions [14]. Survival outcomes for CRC are closely related to the cancer stage at diagnosis [15], and thereby it is one of the few cancers where screening is considered a key preventive measure [16]. A growing number of population-based studies globally have been closely monitoring CRC incidence. Yet, fair comparisons of CRC incidence estimates between different data sources or countries depend on the methods used, which must be explicitly reported.

This article aims to systematically review population-based studies using cancer registries to measure CRC incidence, summarize and describe variation in the commonly employed incidence measures, and evaluate the quality of reporting incidence methods. Our review was set up to answer the following questions: 1- What are the most reported incidence measures for estimating CRC incidence?; 2- What standard populations are commonly used to estimate the age-standardized rate in population-based studies?; 3- Are CRC incidence rates commonly stratified by anatomical site?; 4- What is the quality of reporting the methods used to estimate CRC incidence?

## Methods

The reporting of this systematic review followed the Preferred Reporting Items for Systematic Reviews and Meta-Analyses (PRISMA) [17].

### Study identification

We developed a search strategy in consultation with an information specialist. The search included keywords and a combination of subject headings incorporating “colorectal cancer,” “incidence,” “trends,” and “registry” (the complete search strategy is provided in Additional file 5). We limited the search to articles written in English and to studies published from 1 January 2010 to 31 May 2020. Adding a time frame to the search strategy helped select the most up-to-date studies. The electronic literature search included Embase, Medline, Web of Science, and the Cochrane Library. We also checked reference lists of identified articles for identification of additional potentially relevant articles missed.

### Study selection

Studies were eligible for inclusion in this review based on the inclusion and exclusion criteria presented in Table 1.

**Table 1:** Inclusion and exclusion criteria employed

Inclusion criteria	**•** Population-based retrospective studies using registry data to measure and report the incidence of colorectal cancer **•** English language **•** Full text published **•** Published in a peer-reviewed journal
Exclusion criteria	**•** Studies exclusively measuring the incidence of benign tumours **•** Studies measuring incidence of multiple cancer types **•** Studies reporting incidence measures from external resources **•** Published commentaries **•** Case studies, clinical trials, case–control studies, reviews **•** Conference proceedings, abstracts, posters • Studies conducted in selected population groups (i.e., incidence rates amongst patients with specific diseases)

### Selection process

**Fig. 1:**
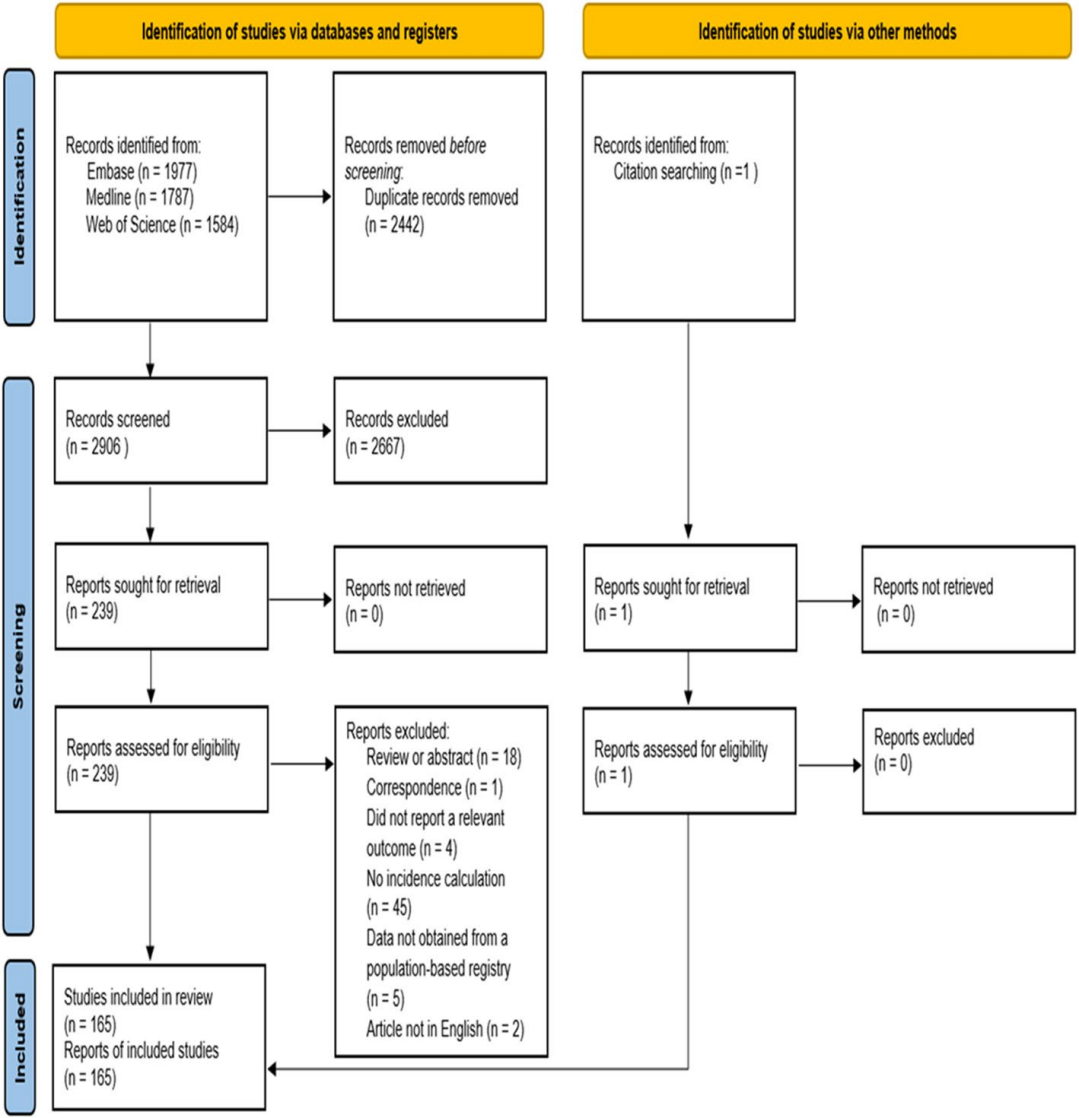
PRISMA flowchart of the study selection process

We imported all potential abstracts into the web app “Rayyan” (a screening software) [18], and two independent reviewers screened all titles and abstracts using the inclusion–exclusion criteria. Any disagreements between reviewers were resolved through discussion. If a consensus decision was not reached by screening the title and abstract, the reviewers examined the full text. We calculated the inter-reviewer agreement rate for title/abstract screening using Cohen’s *κ* statistic (results are presented in Additional file 3: Table 3.1). After the screening process, we further assessed articles selected for full-text review. In cases where eligibility was unclear, we consulted a third reviewer for a final decision. Details of the selection process are displayed in Fig. 1.

### Data extraction and synthesis

We developed and piloted two standardized extraction forms. Form A was used to extract general details about the study, including author and publication year, country, cancer type, main study outcomes, observation period, measures of incidence rate, and the anatomical site used in incidence calculation. Form B was for extracting data necessary to assess the quality of reporting the methods used to calculate incidence. We defined a list of potential indicators to evaluate the reporting quality based on relevant literature on incidence calculation [1–3] and the Guidelines for Accurate and Transparent Health Estimates Reporting (GATHER) statement for reporting global health estimates [6]. This criteria list included: the quality of cancer registry data, the definition of CRC, definition of the numerator, estimation of the denominator, the time interval over which incidence was calculated, presentation of incidence rates, standardization process of rates, age bands for measuring incidence, assessment of uncertainty, evaluation of missing data, and software information. A detailed description of each of these criteria is provided in Table 2. One reviewer extracted the data for all included studies, and a second reviewer cross-checked a random sample of 25% (*n* = 41). Discrepancies were resolved by consensus agreement.

**Table 2:** Criteria for assessing the quality of reporting incidence methods

Criterion	Description
Quality of cancer registry data	The extent to which each study reported details about the quality of the cancer registry data
Definition of colorectal cancer	Report the following: • The used classification system to define CRC • CRC codes (including topography (anatomical site) and morphology (histology, behavior, and grade) codes) • Conversion of ICD codes, if needed • Type of cancer (primary/secondary)
Definition of the numerator	Report any restrictions on included CRC cases
Definition of the denominator (population at risk)	Report the following: • The data source for the general population • The used census years in estimating the at-risk population • The methods used for obtaining postcensal and intercensal population estimates • The estimation of annual mid-year population • The calculation method for estimating average population size over several years of observation
Age-standardized rates (ASRs)	Report the following: • The standardization method used to calculate age-standardized rates (ASRs) • The standard population used in the analysis and why this standard was chosen
The time interval over which incidence is calculated	Report the time interval over which incidence is calculated (e.g., annual, overall average)
Presentation of incidence rates	Incidence rates are expressed with a time unit (whole years or person-time)
Age bands for measuring the incidence	Report the age bands used for measuring and documenting incidence
Assessment of uncertainty	Report the 95% confidence intervals for the Incidence rate
Assessment of missing data	Report missing data assessment and analysis
Software information	Report Software information in the manuscript

### Quality assessment

We appraised the quality of all included studies using a prespecified checklist adapted for this review and based on the Joanna Briggs Institute Critical Appraisal tool for prevalence studies [19] and the Appraisal tool for Cross-Sectional Studies (AXIS) [20]. Both of these tools were previously employed in a systematic review assessing CRC incidence rates [21]. We chose relevant criteria from each tool to create a 10-item checklist for this study. Items were assigned a score of 1 if “demonstrated in the study” or 0 if “not demonstrated or unclear”. We calculated and presented an overall quality score for each study. Quality appraisal checklist and results of quality assessment are presented in Additional file 4.

### Data analysis

The characteristics of included studies, incidence methods, and the quality of reporting incidence were all described in tables. We used descriptive summary statistics to analyse the extracted data and reported the results as frequencies and percentages.

## Results

The combined search initially yielded 5,348 papers, and after the deletion of duplicates, we identified and screened 2906 titles. The inter-reviewer agreement for the title/abstract screening had a Cohen’s *κ* value of 94% (Additional file 3: Table 3.1). After applying the inclusion–exclusion criteria,165 titles were deemed eligible for the systematic review. Details on excluded reports are depicted in the PRISMA flow diagram (Fig. 1).

### Characteristics of included studies

The eligible articles comprised studies from North America, including the United States of America (USA) (*n* = 66, 40%) and Canada (*n* = 5, 3%), Oceania (*n* = 7, 4%), Europe (*n* = 38, 23%), Asia (*n* = 36, 22%), Africa (*n* = 5, 3%), Central and South America (*n* = 2, 1%), and six (4%) multi-country study. In addition to incidence, the two other study outcomes most commonly reported were mortality (*n* = 41, 25%) and survival (*n* = 36, 22%). Most studies evaluated the incidence of colorectal cancer (*n* = 160, 97%), while the remaining evaluated the incidence of either rectal or colon cancer (*n* = 5, 3%). All studies reported the observation period over which incidence was calculated. The periods covered ranged from a single year (*n* = 5, 3%) to 55 years of observation, and 79% covered a study period of ten years or more. The characteristics and details of included studies [22–187] are provided in Additional files 1, 2, 3.

### Measures of incidence rate

The most commonly reported measure of incidence was the age-standardized incidence rate (ASR) (*n* = 132, 80%), followed by the age-specific incidence rate (ASIR) (*n* = 50, 30%), and the crude rate (CR) (*n* = 31, 19%). Five studies reported the calculation of the ASIR but did not present the results of this analysis in the manuscript [22, 30, 34, 36, 62]. The cumulative incidence rate and cumulative risk were reported in three and seven studies, respectively. Some studies also reported the truncated ASR (*n* = 3, 2%), the delay-adjusted rate (*n* = 4, 2%), and the risk-adjusted rate (*n* = 1, 1%) (Table 3).

**Table 3:** Description of the types of measures used for reporting incidence

Incidence measure (as reported)^a^	Definition [1, 90, 133]	*N* (% out of 165)
Age-standardized incidence rate (ASR)	A weighted average of the age-specific incidence rate (weights are from a standard population)	132 (80.0)
Age-specific incidence rate (ASIR)	The number of new cases in a specific age group divided by the corresponding person-years of observation in that particular age group, multiplied by a constant	50 (30.3)
Crude incidence rate (CR)	The number of new cancer cases divided by the total number of person-years of observation, multiplied by a constant	31 (18.8)
Cumulative incidence rate	The total age-specific incidence rate for each year during a specific age span (commonly expressed as a percentage)	3 (1.8)
Cumulative risk	The probability of developing cancer within a specific age span (usually between 0–74), in the absence of competing causes of death (calculated by a formula using the cumulative rate)	7 (4.2)
Truncated ASR	The ASR calculation is restricted to a specific age range (usually 35–64)	3 (1.8)
Delay-adjusted rate	The incidence rate is corrected for the lag in case capture, which affects recent data years	4 (2.4)
Risk-adjusted rate	The numerator in the rate calculation is adjusted for secondary cancers of the same site, and the denominator is adjusted for prevalent cases	1 (0.6)
Incidence rate:	The number of new disease cases in a specific population divided by the population’s size at risk during a particular period	18 (10.9)
• Derived from modelling		4
• Reported as the frequency of new cases		2
• Reported as the percentage of CRC cases among various groups		2

^a^Some studies reported more than one incident measure

Eighteen studies (11%) reported the incidence rate with no further specification. Two of these studies described the incidence as mainly the frequency of new cases [77, 150], four obtained incidence rates via linear modelling [32, 41, 48, 159], and two defined incidence as the percentage of CRC cases among different age groups [119, 120]. Additional details are provided in Additional files 1, 3: Table 3.2.

### The standard population for calculating the age-standardized rate

The 2000 U.S. standard population was the most commonly reported reference population (*n* = 52, 39%), mainly by studies from the USA. The World standard population developed by the World Health Organization (WHO) was the second most reported reference population (*n* = 27, 20%), followed by the European population (*n* = 23, 17%), and the Segi standard population (*n* = 16, 12%)-an older version of the World standard population. Table 3.3 in Additional file 3 provides further details on the reported standard populations used in calculating the ASR.

Of the 127 studies that reported a standard population for ASR estimation, 64 (50%) reported a local reference, 71 (56%) employed an external standard, and four (3%) used both a local and an external standard population [65, 82, 143, 181].

All studies that aimed to conduct international comparisons of ASRs used an external reference population (*n* = 13); three however compared their ASRs with studies that used a different standard population for measuring ASR [44, 97, 175]. Among studies that used standardized rates to assess local incidence rates (*n* = *114*), 62 (54%) employed a local reference, while 52 (46%) used an external standard population.

### Stratification of incidence rate by anatomical site

This review noted that 54% of the 160 identified studies that reported CRC incidence stratified rates by anatomical site. There were variations in terms of the anatomical sites chosen. Of the 86 studies that reported incidence stratified by anatomical location, 77 (90%) stratified rates according to the site (colon/rectum), 33 (38%) by colon site (proximal/distal), and 11 (13%) by the categorization of CRC into “right-sided” or “left-sided” tumour. Seven studies (8%) reported the incidence rate for multiple anatomical sites within the colon, and only four (5%) reported the anus incidence. Details on the anatomical sites used for CRC incidence stratification are provided in Additional files 1, 3: Table3.2.

### The quality of reporting incidence

Table 2 describes the 11 criteria employed to assess quality of incidence reporting. Detailed results for all indicators are provided in Additional files 2–3: Table 3.2.

#### The quality of cancer registry data

Eight studies (5%) reported indicators of data validity, such as the proportion of morphologically verified cases (MV%), percentage of death certificate only cases (DCO%), and mortality to incidence ratio (M/I). Of these studies, five reported estimates for at least one of these indicators [62, 82, 109, 148, 181], and three reported estimates based on external references [139, 173, 184].

Ten studies (6%) cited a reference for previously conducted research as evidence of cancer registry data quality (8 referenced studies or reports including validation or completeness assessments; 2 referenced similar epidemiological studies conducted in the same data source). Six studies (4%) reported that data quality was checked by a cancer registration program such as CanReg4 and CANREGT, but none of these studies provided further details on their inspection results. Singh et al. [154] indicated complete case ascertainment of cancer data used to estimate incidence, but without referencing a specific study. Nine studies (6%) indicated that registration quality was being audited by a certification body. Seven reports (4%) stated that the cancer registry was meeting or utilizing standards for data quality set by national or international agencies. None of these studies provided details on specific quality indicators.

#### The definition of colorectal cancer

There were variations in how studies defined CRC. Only 31 studies (19%) reported whether primary or secondary cancers were considered in the incidence calculation. Forty-five articles (27%) failed to report the classification system used to determine CRC, and 63 (38%) did not provide information about the CRC codes considered. Some studies (*n* = 32, 19%) failed to report both the classification system and CRC codes. In terms of CRC coding, six studies reported only morphological codes, 11 topography and morphology codes, and 85 only topography codes. Furthermore, only 28 articles (17%) explicitly stated whether malignant or in situ cancers were included in the incidence analysis.

Among the studies reporting the classification system used (*n* = 120*,* 73%), the third revision of the International Classification of Disease for Oncology (ICD-O) was the most commonly reported (*n* = 63, 53%) to define CRC, followed by the 10^th^ revision of the International Statistical Classification of Diseases and Related Health Problems (ICD) (*n* = 40, 33%).

Of the 40 studies using the ICD-10 classification system, thirteen (33%) included data from years that preceded its development in 1992. Most of these 13 studies (*n* = 12) failed to document whether they used a different classification system for earlier years or if they mapped codes. Only Wu et al. [174] reported converting earlier ICD codes into those used in the 10^th^ revision. Similarly, of the 63 articles that used the ICD-O-3^rd^ edition, 23 (37%) included data from years that preceded the development of the 3^rd^ or even the 2^nd^ edition of ICD-O with no information provided about conversion of earlier codes.

#### Definition of the numerator

Concerning the reporting of the numerator data, only 20 studies (12%) explicitly explained excluding certain CRC cases from the numerator. The exclusion of non-microscopically confirmed cases [25, 35, 50, 64, 68, 108, 110, 166], and in-situ cancers [54, 65, 68, 110, 145] were the most reported. Details on other restrictions for included CRC cases are provided in Additional file 3: Table 3.2.

#### Definition of the denominator

Concerning reporting of denominator size estimation, over half of the studies failed to state the source of population data used to analyse incidence (*n* = 100, 61%). Only one study explained the calculation used to estimate the annual mid-year population [174]. Twenty-four (15%) of the 165 identified studies indicated the census years employed to derive population counts. Ten studies (6%) reported the method used to estimate yearly population counts (i.e., interpolation or extrapolation).

Only five studies (3%) explicitly explained the population size estimation procedure in calculating the overall average incidence rate (for a given study period). Of these, one study calculated actual person-time at risk by creating closed cohorts of the population on various census nights and following them over time [126]. Three estimated the average population size by multiplying the population count in a particular census year by the number of years included in the study [54, 156, 157]. Sammour et al. [118] estimated the denominator size by averaging population counts of two censuses conducted at the beginning and near the end of the study period.

#### Estimation of the age-standardized rate

Of the studies that calculated the ASR (*n* = 132, 80%), 36 (27%) described the method used for standardization (direct or indirect), with the direct method being the only one reported. Five studies did not report the standard population used to derive ASR.

Of the 127 studies that reported the reference population used for standardization, only five (4%) justified their chosen standard population. Four studies explained that choosing an external (international) reference population will enable future comparisons of incidence rates with other published studies [25, 45, 49, 177]. Jayarajah et al. [83] reported using the WHO World standard population due to its similarity to the age structure of Asian populations.

#### Time interval and presentation of the incidence

Concerning the time interval over which incidence was calculated, over half the studies (*n* = 103, 62%) did not explicitly report whether they calculated a single year or an overall average rate. Assessing how incidence rates were expressed among all included studies, we noted that most articles (*n* = 119, 72%) expressed rates without a time unit (i.e., whole years or person-time).

#### Age bands for measuring the incidence

Among the 165 identified studies, the majority (*n* = 131, 79%) reported the age bands used to calculate incidence. The age bands used for calculating incidence ranged from one (*n* = 12) to 33 (*n* = *1*). Detailed information on all reported age bands is provided in Additional file 3: Table 3.2.

#### Assessment of uncertainty and evaluation of missing data

Concerning uncertainty analysis, only 20% (*n* = 33) reported the confidence interval (CI) associated with the incidence estimate. In examining the reporting of missing data (MD), seven studies (4%) reported details on how MD were handled in the analysis but failed to report assumptions on the reasons for the MD. Of these studies, five reported excluding incident cases with specific MD [53, 92, 99, 111, 114], another study estimated MD by multiple imputation [96], and Zorzi et al. [184] estimated missing variables via join point regression. Missing data in these studies included demographics (such as age, sex, race, country of residence), anatomic subsites, disease stage, and the number of incident cases for some of the years evaluated. One author assumed MD was missed at random, with no justification for this assumption or treatment method reported [115]. Rejali et al. [151] vaguely indicated that incidence rates were corrected for the missing age-related data. Only two studies reported the exact amount of MD [96, 151].

#### Software information

More than half of the studies (*n* = 110, 67%) reported the software used for incidence rate analysis. The most common was The Surveillance Epidemiology and End Results (SEER) statistical software (36%) [188], mainly by studies from the USA. Other reported software included SAS (17%) [189], STATA (16%) [190], and SPSS (16%) [191] (Additional file 3: Table 3.2).

## Discussion

To our knowledge, this is the first study to examine variations in the methods employed in calculating incidence rates and the quality of reporting these methods. The 165 articles retrieved provided valuable findings and insights that will aid future investigators in making informed decisions about which methods and reporting practices will enhance the quality and comparability of their research.

### Measures of incidence rate

Incidence is an essential measure in epidemiology that examines the burden of a disease in a population and highlights variations among different population subgroups. Therefore, incidence measures are imperative for underscoring health care needs and developing policies and interventions accordingly. This review noted that the age-standardized rate (ASR) was the most commonly reported measure of incidence. Only one-third of the studies examined the age-specific incidence rate (ASIR).

ASR is an artificial rate that facilitates comparative analysis as it controls for differences in the population age structure. Relying only on ASRs to describe incidence might conceal valuable information. Thus, the ASIR should always be the starting point when researchers want to derive an accurate measurement of cancer risk in a population [2, 192]. Because ASIRs do not always have a consistent pattern over time, researchers should evaluate patterns of age-specific rates before applying standardization. This analysis would help determine how rates change over time in certain age groups and highlight any irregular patterns requiring further investigation. Furthermore, when possible, researchers should also assess potential effects of birth cohorts (exposures/experiences that vary from one generation to the next) and period (external factors that affect all age groups similarly at a specific calendar time) on age-specific trends [193]. Thus, after initially calculating and graphically presenting the ASIR for different periods or cohorts, regression analysis could be employed to disentangle the effects of age, cohort, and period. This type of analysis however can only be performed when appropriate data is available for long time periods.

In addition to the ASIR, the cumulative rate (calculated using the ASIR), which is a form of standardization not requiring an arbitrary standard population, could be calculated to understand the life-time risk of developing cancer.

In calculating the ASR, only 12 studies, in addition to studies from the USA, employed a local reference, and no study used an internal standard population (the average age distribution of all groups studied). The selection of a standard population is somewhat arbitrary and depends on the study's goals [194]. When the aim is to assess temporal patterns of incidence in a specific population, it is vital to carefully choose a standard that better reflects the study population’s age distribution. On the other hand, when the goal is to compare rates between different populations, an international standard might better serve this purpose [192]. This review noted that most studies failed to justify the selected standard population used to assess CRC incidence. External standards were the most commonly reported, even when a study’s goal was not to compare rates internationally.

The selected standard population can influence the interpretation of incidence. Thus, studies with no international focus but an intention to assess temporal trends could use an internal standard population or employ the base-year population at the start of the study period as the standard [192]. Conversely, if a study aims to compare incidence rates between different countries, conventional external standard populations, such as the WHO World standard [194] and the European standard [195], could be used. To facilitate international comparisons, the WHO emphasized implementing the new revised World standard population, proposed in 2001, reflecting the average age structure of all populations [194]. Using the most updated and appropriate standard population is essential for a more accurate and updated representation of rates. This review noted that 16 studies employed an older version of the World standard- proposed by Segi in 1960- although the new WHO standard was a better fit given the observation study period. Likewise, the European standard (presented in 1976) was employed in six studies where a newer version was available.

Among studies from the USA, a common practice was to standardize rates using the 2000 US standard population. Although this usage is understandably justified, international comparisons with USA rates would be compromised. Meaningful comparisons between populations are only possible when the same reference population is employed. Therefore, investigators could report different ASRs computed by distinct standard populations (an external and the study’s local population) for comprehensive incidence analysis.

In cancer epidemiology, providing incidence estimates according to cancer subsite may highlight critical differences in disease risk. This review noted a lack of consensus concerning the categorization of anatomical subsite for measuring CRC incidence. While almost half of the studies reported only an overall incidence measure for CRC, the other half provided rates according to different categorizations of anatomical sites (e.g., colon/rectal, proximal/distal colon, and right/left colon). Furthermore, descriptions of these anatomical categories varied across studies. Ideally, there should be a consensus among the scientific community on which CRC subsites to consider and on the anatomical categories. Using a standard definition will guide future researchers in reporting comparable incidence rates.

Additionally, when the main study aim is to quantify CRC burden, reporting overall CRC incidence in addition to site-specific rates would facilitate comparison with other studies and evaluation of time trends. Yet, due to data limitations, it might not be feasible for some researchers to include specific subsites in the analysis or to measure site-specific rates. Clarifying these limitations would help the reader better understand the chosen analytical approach.

### The quality of reporting incidence

This review uncovered several limitations in the quality of reporting incidence methods. There was a substantial deficit in reporting registry-data quality control procedures and findings. Population-based cancer registries (PBCR) play a unique role in monitoring and evaluating cancer control efforts. In measuring incidence, PBCR captures all cancer cases in a specified geographical area (numerator) and retrieves population statistics (denominator) from census data. To provide reliable information on cancer burden, it is of utmost importance to ensure that the data are valid and of good quality.

In 1994 The IARC published a report describing standards and methodologies for evaluating data quality in cancer registries [196]. In 2009, two articles updated and summarised these methods in terms of four primary standard indicators: comparability, timeliness, completeness, and validity [5, 9]. This review noted that no study reported details about timeliness issues although many publications didn’t cover recent years in their observation, which may be related to data collection and reporting delays in the registry.

Despite WHO advocacy for strengthening cancer registries, according to the last volume of Cancer Incidence in Five Continents [197], only 65 of 194 WHO Member States provided high-quality cancer incidence data. The proportion of high-quality cancer registries included in the report was 100% in Oceania, 97% in North America, 88% in Europe, 69%, 53%, and 23% in Central and South America, Asia, and Africa, respectively. Additionally, there were considerable discrepancies in total population coverage between continents. Transparent reporting and presentation of quality indicator measures and any registry limitations are essential for accurate interpretation of cancer incidence.

This review noted insufficient reporting of CRC definitions in terms of classification system, codes, and cancer type (primary or secondary tumours). More authors relied solely on topography codes and ignored the importance of reporting the morphology of CRC cases included.

There were also discrepancies across studies concerning the anatomical sites included in CRC incidence calculation; thus, it is essential to comprehensively describe the codes used to define CRC. We also noted limitations in reporting codes conversion between different classification versions. The SEER program and the IARC provide tools to facilitate ICD code mapping between different versions [198, 199]. Authors should clearly document any code conversion implemented. Concerning cancer type, the IARC has set international rules for defining cancer cases as primary or secondary [200]. Cancer registries should use these rules to describe cancer or explicitly acknowledge situations where obtaining this information is not feasible.

Furthermore, this review revealed limitations in reporting the numerator and denominator data used for incidence calculation (e.g., excluding certain CRC cases from the numerator). Being explicit about such information is valuable for interpreting and comparing rates. However, it is important to note that cancer registries differ in the type of data collected. For example, some registries collect data regarding hereditary syndromes or risk factors for CRC, while others do not. Limitations in terms of data availability should be acknowledged.

More than half of the studies included in this review presented an overall average incidence measure although only five articles described the calculation of the total population estimate (over several years of observation). Furthermore, there was an evident lack in reporting the source of population data, how yearly mid-year population statistics were estimated, and the census years used for obtaining population statistics. In analysing incidence rates, describing the estimation of the denominator is usually overlooked, especially when calculating the ASR. Although the standardization process controls for the effect of population age structure, some might understand this process as eliminating the impact of population structure on incidence rates [2]. Population size estimations used as denominators have their limitations that authors should explicitly recognize. Explaining how these estimates were derived is essential for understanding cancer risk and ensuring that readers have sufficient details to reproduce the findings.

The results of this review also highlighted other deficiencies in reporting incidence rates, such as the indication of the time interval over which incidence is calculated, the expression of rates, and the reporting of uncertainty estimates. In terms of quantifying and reporting uncertainty around incidence estimates, we noted that only 33 studies reported CI. Population-based studies tend to underestimate the importance of reporting CIs for estimates drawn from population-level data [201]. For some researchers, the observed rates represent accurate measurements for the population rather than estimates, and thus, accounting for random error might not be needed. However, Redelings et al. [201] argue that as rates and trends tend to fluctuate randomly over time, due to a myriad of factors, reporting the CI is imperative for assessing the reliability of these estimates and will consequently aid the formation of public health interventions and policies [201].

This review also highlighted inadequate reporting of MD analysis, including assumptions on the reasons for the missingness and their justification, the amount of MD, and the methods used to handle them in the analysis. Reporting guidelines for observational studies emphasized the need for a complete and transparent reporting of missing data and its analysis [6, 202]. Thus, researchers should explicitly acknowledge and document all details pertaining to MD analysis.

### Strengths and limitations

To our knowledge, this study is the first to comprehensively review the methods employed to estimate incidence rate and the degree of quality and transparency in their reporting. The identified studies were conducted in different populations and settings. We used multiple indicators for quality assessment, based on relevant literature on incidence calculation and guidelines for reporting methods, which enriches the evidence provided in this review. Although detailed reporting of methods might sometimes be limited by journal policies (i.e., word count restrictions), information could be made available as supplemental or web-based data.

This review is limited to studies assessing incidence in CRC using registry data. Despite this, our results inform about the most commonly used measures of estimating disease incidence and provide general considerations for improving the quality of reporting for other cancer types or diseases.

Another limitation was limiting the search to articles published within the past decade to limit the scope of the review. Given that there have been no substantial changes to the measures used for estimating incidence rates, we believe that the time-frame restriction did not affect the findings. Although we searched multiple databases and included studies from different countries, we included only English articles in this review. Thus, we might have missed relevant papers in other languages.

### Future research

This review highlighted variations in reporting standards despite continuous efforts by scientific organizations, such as the EQUATOR Network, to provide guidance to help achieve an acceptable standardized level of reporting.

The GATHER statement promotes good quality reporting of global health estimates by providing a list of items that should be described when reporting health estimates [6]^.^ Our review emphasized reporting some of the GATHER items relating to the study’s methodology, including data source, the uncertainty of estimates, handling missing data, and software package. This study, however, recommends other areas for consideration when reporting incidence measures. Future research on disease incidence should comprehensively describe their methodology based on these recommendations. We hope this study will be the starting point toward developing a specific guideline for reporting disease incidence in large-population studies.

## Conclusion

This review summarized the most commonly reported incidence measures and examined variations in estimating CRC incidence over the past decade. We also highlighted many deficiencies in incidence reporting and provided recommendations for future studies on how to optimize their communication of the methods used for estimating incidence. Ideally, reporting should provide sufficient detail on the methodology to enable replicating the analysis. Better reporting will facilitate interpreting and comparing results with other studies and help identify and address limitations of the analysis.

## Supplementary Information

**Additional file 1.** Data extraction sheet (form A).

**Additional file 2.** Data extraction sheet (form B).

**Additional file 3.** Results tables.

**Additional file 4.** Quality assessment.

**Additional file 5.** Search strategy.

## Data Availability

All data generated and analysed during this study are included in this article and its supplementary information files.

## Abbreviations

ASIR: Age-specific incidence rate
ASR: Age-standardized rate
AXIS: Appraisal Tool for Cross-Sectional Studies
CI: Confidence interval
CR: Crude rate
CRC: Colorectal cancer
DCO: Death certificate only
EQUATOR: Enhancing the Quality and Transparency of Health Research
GATHER: Guidelines for Accurate and Transparent Health Estimates Reporting
IARC: International Agency for Research on Cancer
ICD: International Statistical Classification of Diseases and Related Health Problems
ICD-O: International Classification of Disease for Oncology
MD: Missing data
M/I: Mortality to incidence ratio
MV: Morphologically verified
PRISMA: Preferred Reporting Items for Systematic Reviews and Meta-Analyses
PBCR: Population-based cancer registries
SAS: Statistical Analysis System
SEER: Surveillance Epidemiology and End Results
SPSS: Statistical Package for The Social Sciences
USA: United States of America
WHO: World Health Organization
