# Supplementary material for: A systematic review of methods to estimate colorectal cancer incidence using population-based cancer registries
Source: BMC Med Res Methodol. 2022 May 19;22:144. doi: 10.1186/s12874-022-01632-7 (PMC9118801; doi:10.1186/s12874-022-01632-7)
Supplement: Supplementary file 1 — Additional file 1. Data extraction sheet (form A). [file 12874_2022_1632_MOESM1_ESM.docx]

**Additional file 1** Data extraction sheet (form A) (P.1-14)

| **Table 1.1** Characteristics of included studies, measures of incidence, and chosen anatomical site for reporting incidence | | | | | | | |
| --- | --- | --- | --- | --- | --- | --- | --- |
|  | **First author and year** | **Country** | **Cancer type** | **Main outcomes** | **Observation period** | **Measure/s of incidence** | **Anatomical site stratification** |
| 1 | Abdifard 2016 | Iran | CRC | Incidence | 2000–2009 | ASR, ASIR | CRC |
| 2 | Abdifard 2013 | Iran | CRC | Incidence | 2000–2005 | ASR | CRC |
| 3 | Abreu 2010 | Portugal | Rectal | Incidence, survival | 1995–2004 | Sex and Age standardized incidence rate, CR, Cumulative risk | Rectum |
| 4 | Hassan 2016 | Malaysia | CRC | Incidence, mortality | 2008–2013 | ASR | CRC |
| 5 | Abualkhair 2020 | USA | CRC | Incidence | 2000–2015 | ASR | (Colon, rectum, CRC) |
| 6 | Al Dahhan 2018 | Iraq | CRC | Incidence | 2002–2011 | CR | CRC |
| 7 | Araghi 2018 | USA | CRC | Incidence, projection | 1973–2014 | ASR | CRC |
| 8 | Araghi 2019 | Seven high income countries | CRC | Incidence | 2008–2012/2009–2013/ 2010–2014 | ASR | (Colon, rectum) |
| 9 | Ashktorab 2016 | USA | CRC | Incidence | 2000–2012 | ASR, ASIR | CRC |
| 10 | Austin 2014 | USA | CRC | Incidence | 1998–2009 | ASR | (Proximal/distal colon, rectum, CRC)^A^ |
| 11 | Aziz 2015 | USA | CRC | Incidence | 1995–2010 | Mean incidence from modelling | CRC |
| 12 | Bailey 2015 | USA | CRC | Incidence | 1975–2010 | ASR | (Colon, right/left colon, rectosigmoid and rectum, colon (NOS)) |
| 13 | Baniasadi 2015 | Iran | CRC | Incidence | 2003–2013 | ASR, ASIR | CRC |
| 14 | Bhurgri 2011 | Pakistan | CRC | Incidence, gender and clinical distribution | 1995–1997/ 1998–2002 | CR, ASR, ASIR | (Colon, rectum and anus, CRC) |
| 15 | Boyce 2016 | Australia | CRC | Incidence, clinical and demographic features, survival | 2001–2008 | ASR, ASIR | CRC |
| 16 | Winther 2016 | Denmark | CRC | Incidence, prevalence, survival, mortality | 1980–2012 | ASIR | (Colon, rectum and anus) |
| 17 | Brenner 2016 | Germany | CRC | Incidence, mortality | 2003–2012 | ASR, Cumulative risk | CRC |
| 18 | Brouwer 2018 | Netherlands | CRC | Incidence, mortality, treatment, survival | 1989–2014 | ASR | (Colon, rectum) |
| 19 | Caldarella 2013 | Italy | CRC | Incidence | 1985–2005 | ASR, ASIR | (Proximal/distal colon, rectum, CRC)^A^ |
| 20 | Carroll 2019 | USA | CRC | Incidence, survival | 1973–2013 | Incidence rate from Poisson modelling | CRC |
| 21 | Chambers 2020 | United Kingdom | CRC | Incidence | 1974–2015 | ASR, ASIR | (Proximal/distal colon, CRC)^B4^ |
| 22 | Chatterjee 2015 | USA | CRC | Incidence, screening practices, CRC risk factors | 2000–2009 | ASR | CRC |
| 23 | Alsanea 2015 | Saudi Arabia | CRC | Incidence, survival, demographic features | 1994–2010 | ASR | CRC |
| 24 | Chauvenet 2011 | France | CRC | Incidence | 1976–2005 | ASR, Cumulative risk | (Right/left colon, sigmoid, rectum, CRC) ^C^ |
| 25 | Chen 2012 | Taiwan | CRC | Incidence | 1988–2007 | ASIR | (CRC, left/right colon, colon, rectum)^D^ |
| 26 | Cheng 2011 | USA | CRC | Incidence | 1976–2005 | ASR | (Proximal/distal colon, rectum) ^A^ |
| 27 | Chernyavskiy 2019 | USA | CRC | Incidence | 2000–2014 | Incidence rate from modelling | (Colon, rectum) |
| 28 | Chittleborough 2020 | New Zealand, Sweden, and Scotland | CRC | Incidence | 1995–2012/1970–2014/ 1990–2014 | ASR | (Rectum, colon, distal/proximal colon, CRC)^A^ |
| 29 | Chong 2015 | Brunei Darussalam | CRC | Incidence | 1991 and 2014 | ASR, ASIR | (Rectum, colon, CRC) |
| 30 | Clarke 2014 | Ireland | CRC | Incidence, age and stage distribution, treatment, mortality, survival | 1994–2010 | ASR | (Colon, rectum, CRC) |
| 31 | Crocetti 2010 | Italy | CRC | Incidence | 1985–2005 | ASR | CRC |
| 32 | Crosbie 2018 | USA | CRC | Incidence, demographic and clinical features | 1979–2014 | ASR | (Proximal/distal colon, rectum, CRC)^A1^ |
| 33 | Danos 2018 | USA | CRC | Incidence | 2008–2012 | CR | CRC |
| 34 | Dehghani 2019 | Iran | CRC | Incidence | 2003–2010 | CR, ASIR | CRC |
| 35 | Edwards 2010 | USA | CRC | Incidence, mortality, survival, projection | 1975–2006 | ASR, ASIR | CRC |
| 36 | Ellis 2018 | USA | CRC | Incidence | 1990–2014 | ASR | CRC |
| 37 | Enayatrad 2018 | Iran | CRC | Incidence | 2009 | ASR | CRC |
| 38 | Eser 2018 | Cyprus, Jordan, Israel, and İzmir/Turkey | CRC | Incidence | 2005–2010 | ASR, CR | (Colon, rectum, CRC) |
| 39 | Exarchakou 2019 | England | CRC | Incidence | 1971–2014 | ASR, ASIR, CR | (Right/left colon, rectum, colon (NOS), CRC)^F^ |
| 40 | Feletto 2019 | Australia | CRC | Incidence | 1982–2014 | ASIR | (Colon, rectum) |
| 41 | Hasanpour-Heidari 2019 | Iran | CRC | Incidence | 2004–2013 | ASR, ASIR, CR | (Colon, rectum, CRC) |
| 42 | Lemmens 2010 | Netherlands | CRC | Incidence, stage distribution, treatment, mortality, survival | 1975–2007 | ASR | (Colon, rectum, ascending colon, transverse colon, descending and sigmoid colon) |
| 43 | May 2017 | USA | CRC | Incidence, stage distribution | 1975–2012 | ASR | CRC |
| 44 | Fusco 2010 | Italy | CRC | Incidence, mortality, survival, clinical and demographic features | 2000–2005 | ASR, CR, ASIR, Cumulative risk | CRC |
| 45 | Klugarova 2019 | Czech Republic | CRC | Incidence, prevalence, mortality, treatment, survival | 1982–2016 | Incidence rate | CRC |
| 46 | Koblinski 2018 | USA | CRC | Incidence | 2000–2010 | Incidence rate | CRC |
| 47 | Martinsen 2016 | USA | CRC | Incidence, mortality, survival | 1990–2012 | ASR | (Distal/proximal colon, CRC)^B^ |
| 48 | Giddings 2012 | USA | CRC | Incidence | 1988–2007 | ASR | CRC |
| 49 | Missaoui 2011 | Tunisia | CRC | Incidence | 1993–2007 | ASR, CR, ASIR | CRC |
| 50 | Kelly 2012 | USA | CRC | Incidence | 2005–2009 | ASR, ASIR | (Colon, proximal/distal colon, rectum, CRC)^A^ |
| 51 | Loomans-Kropp 2019 | USA | CRC | Incidence, mortality | 1980–2016 | ASR | (Rectum, colon, proximal/distal colon)^A1^ |
| 52 | Gandhi 2017 | New Zealand | CRC | Incidence | 1995–2012 | ASR, CR | (Proximal/distal colon, rectum, CRC)^A^ |
| 53 | Lopez 2019 | France | CRC | Incidence, management, recurrence, survival | 1982–2011 | ASR | CRC |
| 54 | Lopez-Abente 2010 | Spain | CRC | Incidence, mortality | 1975–1993/2000–2004 | ASR | CRC |
| 55 | McClements 2012 | United Kingdom | CRC | Incidence, stage distribution, mortality | 1982–2006 | ASIR | CRC |
| 56 | Gan 2019 | USA | CRC | Incidence, screening practices, survival | 2011–2016 | Incidence rate (defined as number of CRC cases) | CRC |
| 57 | Ladabaum 2014 | USA | CRC | Incidence | 1990–2004 | ASR | CRC |
| 58 | Fowler 2018 | USA | CRC | Incidence, mortality | 1991–2010 | ASR | CRC |
| 59 | Meester 2019 | USA | CRC | Incidence, stage distribution | 1975–2015 | ASR | CRC |
| 60 | Li 2017 | China | CRC | Incidence | 1998–2012 | ASR, CR | (Colon, rectum, CRC, proximal/ distal colon)^A^ |
| 61 | Liu 2015 | China | CRC | Incidence, mortality | 2011 | ASR, ASIR, CR, Truncated ASR (35-64), Cumulative incidence rate | CRC |
| 62 | Jayarajah 2020 | Sri Lanka | CRC | Incidence, clinical features | 2001–2010 | ASR, ASIR | CRC |
| 63 | Katsidzira 2016 | Zimbabwe | CRC | Incidence, demographic and clinical features | 2003–2012 | ASR, ASIR | CRC |
| 64 | Fournel 2016 | France | CRC | Incidence | 1995–2002 | Sex and Age standardized incidence rate | CRC |
| 65 | Lee 2019 | Taiwan | CRC | Incidence, survival, mortality | 1984–2013 | ASIR | CRC |
| 66 | Khiari 2017 | Tunisia | CRC | Incidence, age and clinical distribution | 2007–2009 | ASR, CR | (Colon, rectum, CRC) |
| 67 | Shadmani 2017 | Iran | CRC | Incidence | 2008 | ASR, CR | CRC |
| 68 | Merrill 2011 | USA | CRC | Incidence | 2005–2007 | ASR, Risk-adjusted incidence rate | CRC |
| 69 | Klimczak 2011 | Poland | CRC | Incidence, prevalence | 1999–2008 | ASR | (Colon, rectum) |
| 70 | Khiari; Ben Ayoube 2017 | Tunisia | CRC | Incidence, projection | 1994–2009 | ASR | (CRC, colon, proximal/distal colon, rectum)^A^ |
| 71 | Jandova 2016 | USA | CRC | Incidence, mortality, demographic and clinical features | 1995–2011 | Incidence rate | CRC |
| 72 | Li; Lin 2017 | China | CRC | Incidence | 2010–2014 | ASR, CR | CRC |
| 73 | Meza 2010 | United Kingdom and USA | CRC | Incidence | 1973–2006 | ASR | (Proximal/distal colon, rectum)^B^ |
| 74 | Jafri 2013 | USA | CRC | Incidence, survival | 1993–2007 | ASR, ASIR | (CRC, Cecum, ascending colon, hepatic flexure, transverse colon, splenic flexure, descending colon, sigmoid colon, Rectosigmoid junction, rectum, and large intestine (NOS)) |
| 75 | McDevitt 2017 | Ireland | CRC | Incidence, mortality, survival, anatomical site and stage distribution | 1994–2012 | ASR | (Colon, proximal/distal colon, overlapping and colon (NOS), rectosigmoid junction and rectum, CRC)^B1^ |
| 76 | Khachfe 2019 | Lebanon | CRC | Incidence | 2005–2015 | ASR, ASIR | CRC |
| 77 | Meyer 2010 | USA | CRC | Incidence | 1973–2005 | ASR | (Rectum, rectosigmoid junction, sigmoid colon, descending colon, colon excluding rectum) |
| 78 | Garcia 2018 | USA | CRC | Incidence | 2001–2014 | ASR, ASIR | (Proximal/distal colon, rectum, CRC)^A1^ |
| 79 | Fournel 2012 | France | CRC | Incidence, stage distribution | 1990–1999 | Sex and Age standardized incidence rate, CR | CRC |
| 80 | Brenner 2017 | Canada | CRC | Incidence | 1971–2012 | ASIR | (Colon, rectum) |
| 81 | Brenner 2019 | Canada | CRC | Incidence | 1971–2015 | ASR, ASIR | (Colon, rectum) |
| 82 | Fedewa 2019 | USA | CRC | Incidence, colonoscopy rate | 2000–2015 | Delay-adjusted incidence rate | CRC |
| 83 | Augustus 2018 | USA | CRC | Incidence | 2000–2014 | ASR | (Proximal/distal colon, CRC)^B3^ |
| 84 | Davis 2011 | USA | CRC | Incidence, age and anatomical site distribution | 1987–2006 | ASIR | (Colon, CRC, cecum, appendix, ascending colon, hepatic flexure, transverse colon, splenic flexure, descending colon, sigmoid colon, rectosigmoid junction, rectum) |
| 85 | Domati 2014 | Italy | CRC | Incidence, survival, clinical features | 1986–2008 | CR | CRC |
| 86 | Koblinski 2019 | USA | CRC | Incidence, demographic and clinical features | 2000–2010 | Incidence rate | CRC |
| 87 | Purim 2013 | USA | CRC | Incidence, survival, stage distribution | 2002–2006 | ASR, ASIR | (Rectum, colon) |
| 88 | Vuik 2019 | Europe | CRC | Incidence, mortality | 1990–2016 | ASR, ASIR | (CRC, colon, rectum) |
| 89 | Shafqat 2015 | USA | CRC | Incidence, survival, management | 2000–2011 | ASR | CRC |
| 90 | Safaee 2012 | Iran | CRC | Incidence | 2005–2009 | ASR | (Colon, rectum, rectosigmoid junction, anus, anal canal, CRC) |
| 91 | Siegel 2017 | USA | CRC | Incidence, mortality, survival, stage distribution, screening prevalence | 2009–2013 | ASR | (Proximal/distal colon, rectum, appendix/ unspecified subsite, CRC)^A^ |
| 92 | Singh 2018 | Canada | CRC | Incidence | 1985–2012 | Sex and Age standardized incidence rate | (CRC, proximal/distal colon)^B1^ |
| 93 | Savijarvi 2019 | Finland | CRC | Incidence | 1976–2014 | ASR | (Colon, proximal/distal colon, rectum, CRC)^B1^ |
| 94 | Rahman 2015 | USA | CRC | Incidence, survival | 1992–2009 | ASR | CRC |
| 95 | Nfonsam 2015 | USA | CRC | Incidence, mortality, stage distribution | 1995–2010 | Incidence rate | CRC |
| 96 | Van Beck 2018 | USA | CRC | Incidence, mortality | 1976–2015 | ASR | CRC |
| 97 | Sammour 2009 | New Zealand | Colon | Incidence, mortality, survival, anatomical site and stage distribution | 1996–2003 | ASR | Colon |
| 98 | Mosli 2012 | Saudi Arabia | CRC | Incidence, clinical features | 2001–2006 | Incidence (presented as percentage) | (Colon, rectum, CRC) |
| 99 | Mosli 2012 | Saudi Arabia | CRC | Incidence, clinical features | 2000–2006 | Incidence (presented as percentage) | CRC |
| 100 | Russo 2019 | Italy | CRC | Incidence | 1999–2015 | ASIR | (Colon, rectum, CRC) |
| 101 | Sheneman 2017 | USA | CRC | Incidence, survival | 1992–2013 | ASR, ASIR | (Left/right colon, CRC) |
| 102 | Oliphant 2011 | United Kingdom | CRC | Incidence | 1999–2007 | ASR | CRC |
| 103 | Perdue 2014 | USA | CRC | Incidence, mortality | 2005–2009 | ASR | CRC |
| 104 | Murphy 2017 | USA | CRC | Incidence | 1975–2013 | ASR | (CRC, proximal/distal colon, rectum)^A1^ |
| 105 | Shah 2012 | New Zealand | CRC | Incidence | 1981–2004 | ASR | (Right/left colon, rectum)^D^ |
| 106 | Siegel 2020 | USA | CRC | Incidence, mortality, screening prevalence, survival, stage distribution | 2012–2016/1995–2016 | ASR, Delay-adjusted incidence rate | (Colon, rectum, CRC, proximal/distal colon, appendix, large intestine (NOS), CRC including appendix)^A^ |
| 107 | Murphy 2011 | USA | CRC | Incidence | 1992–2006 | ASR | (Proximal/distal colon, rectum)^A^ |
| 108 | Shin 2012 | Korea | CRC | Incidence | 1999–2009 | ASR | (CRC, proximal/distal colon, rectum)^B1^ |
| 109 | Patel 2016 | Canada | CRC | Incidence, CRC risk factors | 1969–2010 | ASR | (CRC, colon, rectum and rectosigmoid) |
| 110 | Pakzad 2016 | Iran | CRC | Incidence, spatial distribution | 2009 | ASR | CRC |
| 111 | Siegel; Fedewa 2017 | USA | CRC | Incidence | 1974–2013 | ASR, ASIR, Delay-adjusted incidence rate, | (Colon, rectum, proximal/distal colon)^A1^ |
| 112 | Pescatore 2013 | Luxembourg | CRC | Incidence, survival, stage distribution | 1990–2009 | ASR, CR, ASIR | CRC |
| 113 | Murphy 2018 | USA | CRC | Incidence | 1975–2014 | ASR, ASIR | (CRC, proximal/distal colon, rectum)^A1^ |
| 114 | Siegel 2012 | USA | CRC | Incidence | 1992–2008 | ASR | (Right/left colon)^D1^ |
| 115 | Siegel 2019 | USA | CRC | Incidence, CRC risk factors | 1995–2015 | ASR | (Colon, rectum, CRC) |
| 116 | Sung 2019 | Hong Kong, Korea, Japan, and Taiwan | CRC | Incidence | 1995–2014 | ASR | (Colon, rectum) |
| 117 | Rafiemanesh 2016 | Iran | CRC | Incidence, clinical features | 2003–2008 | ASR, CR | CRC |
| 118 | Sierra 2016 | Central and South America | CRC | Incidence, mortality | 2003–2007 | ASR, CR | CRC |
| 119 | Oliveira 2016 | Brazil | CRC | Incidence, mortality | 1988–2008 | ASR | (Colon, rectum) |
| 120 | Palmieri 2013 | Italy | CRC | Incidence, mortality, survival, demographic and clinical features | 1992–2010 | ASR, CR, ASIR, Cumulative risk | CRC |
| 121 | Paquette 2015 | USA | CRC | Incidence | 2000–2011 | ASR, ASIR | (Colon, rectum, CRC) |
| 122 | Reggiani-Bonetti 2013 | Italy | CRC | Incidence, clinical features | 1986–2008 | ASR, CR | (Colon, rectum, CRC) |
| 123 | Nowicki 2018 | Poland | CRC | Incidence, morbidity, survival | 2006–2011 | CR (refers to the frequency of new cases reported for the first time in a given year) | (Colon, rectosigmoid junction, rectum) |
| 124 | Phipps 2012 | USA | CRC | Incidence, mortality | 1975–2007 | ASR | (Proximal/distal colon, CRC)^A2^ |
| 125 | Oppelt 2019 | Germany | CRC | Incidence | 2008–2014 | ASR | CRC |
| 126 | Murphy 2019 | USA | CRC | Incidence, survival | 1992–2014 | ASR | (Proximal/distal colon, rectum, appendix/unspecifie) ^A1^ |
| 127 | Innos 2018 | Estonia | CRC | Incidence, survival | 1995–2014 | ASR, ASIR | (Colon, rectum, right/left colon, colon other, anus and anal canal)^F^ |
| 128 | Siegel 2014 | USA | CRC | Incidence, survival, mortality, anatomical site and stage distribution | 1975–2010 | ASR, Delay-adjusted incidence rate | (Proximal/distal colon, rectum, colon other, CRC)^A^ |
| 129 | Sia 2014 | Australia | CRC | Incidence, anatomical site and histopathology distribution | 2000–2010 | Incidence rate (defined as number of CRC cases) | (Colon, rectum, CRC) |
| 130 | Rejali 2018 | Iran | CRC | Incidence | 2000–2011 | ASR, Truncated ASR (at 25) | CRC |
| 131 | Sarakarn 2017 | Thailand | CRC | Incidence | 1989–2012 | ASR | CRC |
| 132 | Keum 2014 | USA | CRC | Incidence, mortality | 1975–2009 | ASR, ASIR | CRC |
| 133 | Singh 2014 | USA | CRC | Incidence | 1988–2009 | ASIR | (CRC, proximal/distal colon, rectum)^A^ |
| 134 | Sjostrom 2018 | Sweden | CRC | Incidence, mortality, survival | 2007–2013 | ASR | (Colon, rectum) |
| 135 | Steinbrecher 2012 | USA | CRC | Incidence, mortality | 1998–2002 | ASR | (CRC, right/left colon, rectum)^F^ |
| 136 | Stern 2016 | USA | CRC | Incidence, demographic and clinical features | 1995–2011 | ASR | CRC |
| 137 | Stock 2012 | USA | CRC | Cumulative risk | 1978–2007 | Cumulative incidence rates, Cumulative risk | (CRC, colon, rectum, proximal/distal colon)^A3^ |
| 138 | Stromberg 2019 | Sweden | CRC | Incidence, mortality | 2008–2016 | Incidence rate from Poisson modelling | CRC |
| 139 | Sun 2020 | Sweden | CRC | Incidence, survival | 1960–2014 | Age standardized-sex-specific incidence rate | (Right/left colon, rectum)^E^ |
| 140 | Tawadros 2015 | USA | Rectal | Incidence, clinical features | 1980–2010 | Incidence rate | Rectum |
| 141 | Thirunavukarasu 2010 | USA | CRC | Incidence, survival, clinical and demographic features | 1973–2006 | Incidence rate | CRC |
| 142 | Thuraisingam 2017 | USA | Colon | Incidence | 2000–2012 | Incidence rate | Colon |
| 143 | Troeung 2017 | Australia | CRC | Incidence, mortality, colonoscopy history | 1982–2007 | ASR, ASIR | CRC |
| 144 | Ugarte 2012 | Spain | CRC | Incidence | 1990–2005 | ASR, ASIR | CRC |
| 145 | Ullah 2018 | Ireland | CRC | Incidence, stage distribution | 1994–2012 | ASR | CRC |
| 146 | Veruttipong 2012 | Egypt | CRC | Incidence, clinical and demographic features | 1999–2007 | ASR, ASIR | (Colon, rectum, CRC) |
| 147 | Wan Ibrahim 2020 | Malaysia | CRC | Incidence, mortality, survival, clinical and demographic features | 2007–2017 | ASR, CR | CRC |
| 148 | Wang 2017 | USA | CRC | Incidence, survival, stage distribution | 1995–2010 | ASR | (CRC, right/left colon)^D1^ |
| 149 | Wang; de Grubb 2017 | USA | CRC | Incidence | 1994–2013 | ASR | (Proximal/distal colon, rectum, CRC)^A^ |
| 150 | Wang 2019 | USA | CRC | Incidence, factors associated with cancer-specific death | 1988–2013 | ASR | CRC |
| 151 | Wen 2018 | China | CRC | Incidence | 2012/2000–2015 | ASR, ASIR, CR, Cumulative incidence rate | CRC |
| 152 | Wessler 2010 | Norfolk, Suffolk, Cambridgeshire (NSC)  (East of England) | CRC | Incidence | 1971–2005 | ASR, ASIR, CR | (CRC, proximal/distal colon, colon, rectum)^A^ |
| 153 | Wu 2018 | Shanghai | CRC | Incidence, mortality | 1975–2013 | ASR, CR | (CRC, colon, rectum) |
| 154 | Yee 2010 | Hong Kong | CRC | Incidence | 1983–2006 | ASR, CR, Incidence rate | CRC |
| 155 | Yeo 2017 | USA | CRC | Incidence, clinical and demographic features | 2000–2011 | ASR | (Cecum, ascending colon, hepatic flexure, transverse colon, splenic flexure, descending colon, sigmoid, rectosigmoid junction, rectum) |
| 156 | Yoon 2015 | Korea | CRC | Incidence, mortality, fatality, screening rate | 1999–2012 | ASR, ASIR | CRC |
| 157 | Young 2015 | Canada | CRC | Incidence | 1998–2009 | ASR | (Colon, rectum, CRC) |
| 158 | Zhabagin 2015 | Kazakhstan | CRC | Incidence, mortality | 2004–2013 | Incidence rate | (Colon, rectum, CRC) |
| 159 | Zhang 2018 | Hong Kong | CRC | Incidence | 1983–2012 | ASR | (Colon, rectum) |
| 160 | Zheng 2014 | China | CRC | Incidence, mortality | 2010 | ASR, CR, ASIR, Cumulative risk, Truncated ASR (35-64) | CRC |
| 161 | Zhou 2015 | China/Guangzhou | CRC | Incidence, age and anatomical site distribution | 2000–2011 | ASR, CR, ASIR | (CRC, ascending colon, transverse colon, descending colon, sigmoid colon, rectum) |
| 162 | Zhu 2013 | USA | Colon | Incidence | 1973–2008 | Age standardized-sex-specific incidence rate, ASIR | (Cecum, appendix, ascending colon, hepatic flexure, transverse colon, splenic flexure, descending colon, sigmoid colon, overlapping lesion of colon, colon (NOS)) |
| 163 | Zorzi 2019 | Italy | CRC | Incidence, mortality | 2003–2014 | ASR | (Colon, rectum, CRC) |
| 164 | Zorzi 2015 | Italy | CRC | Incidence | 2000–2008 | ASR | (CRC, colon (NOS), proximal/distal colon, rectum)^B2^ |
| 165 | Ohri 2020 | USA | CRC | Incidence | 2000–2014 | ASR | CRC |

**Abbreviations:** CRC: Colorectal cancer, USA: United States of America, ASR: Age-standardized incidence rate, ASIR: Age-specific incidence rate, CR: Crude incidence rate, NOS: Not otherwise specified.

**Definitions of proximal/distal, right/left tumors:**

- A: Proximal colon: cecum, ascending colon, hepatic flexure, transverse colon, and splenic flexure. Distal colon: descending colon and sigmoid colon.
- A1: Same as (A) but splenic flexure is in the distal colon.
- A2: Same as (A) but rectosigmoid junction and rectum are in the distal colon.
- A3: Proximal colon: cecum, ascending colon, hepatic flexure, transverse colon. Distal colon: splenic flexure, descending colon, sigmoid colon, rectosigmoid junction, and rectum.
- B: Proximal colon: cecum, appendix, ascending colon, hepatic flexure, transverse colon. Distal colon: splenic flexure, descending colon, sigmoid colon.
- B1: Same as (B) but splenic flexure is in the proximal colon.
- B2: Same as (B) but overlapping lesion of the colon is in the distal colon.
- B3: Same as (B) but rectosigmoid junction and rectum are in the distal colon.
- B4: Proximal colon: cecum, appendix, ascending colon, hepatic flexure, transverse colon, splenic flexure, descending colon. Distal colon: sigmoid colon, rectosigmoid junction, and rectum.
- C: Right colon: cecum, ascending colon, hepatic flexure, transverse colon. Left colon: splenic flexure and descending colon.
- D: Right colon: cecum, ascending colon, hepatic flexure, transverse colon, and splenic flexure. Left colon: descending colon, sigmoid colon, rectosigmoid junction, and rectum.
- D1: Same as (D) but splenic flexure is in the left colon.
- E: Right colon: cecum, ascending colon, transverse colon, and splenic flexure. Left colon: descending colon and sigmoid colon.
- F: Right colon: cecum, appendix, ascending colon, hepatic flexure, and transverse colon. Left colon: splenic flexure, descending colon, and sigmoid colon.
