# Supplementary material for: A systematic review of methods to estimate colorectal cancer incidence using population-based cancer registries
Source: BMC Med Res Methodol. 2022 May 19;22:144. doi: 10.1186/s12874-022-01632-7 (PMC9118801; doi:10.1186/s12874-022-01632-7)
Supplement: Supplementary file 2 — Additional file 2. Data extraction sheet (form B). [file 12874_2022_1632_MOESM2_ESM.docx]

| **Additional file 2** Data extraction sheet (form B) (P.1-15) | | | | | | | | | | | |
| --- | --- | --- | --- | --- | --- | --- | --- | --- | --- | --- | --- |
| **Table 2.1** Criteria for assessing the quality of reporting incidence | | | | | | | | | | | |
| **First author and year** | **Definition of CRC** | **Quality assessment of registry data** | **Definition of numerator** | **Definition of denominator** | **Time interval for incidence calculation** | **Presentation of incidence rates with a time unit** | **Age-standardized rates**  (Method/ standard population) | **Age bands** | **Assessment of uncertainty** | **Assessment of missing data** | **Software information** |
| Abdifard 2016 | ICD: site codes provided. | Not reported | CRC cases | C3/C4/C5 | Not clear (mostly annual) | Whole years | (Direct /WHO world standard population) | Seven | Not reported | Not reported | STATA |
| Abdifard 2013 | ICD-O-2: site codes provided. | Not reported | CRC cases | C4/C5 | Not clear (mostly annual) | Whole years | (Direct /WHO world standard population) | Nine | Not reported | Not reported | STATA |
| Abreu 2010 | ICD-O-3 | Not reported | Rectal cancer cases | C4/C5 | Not clear (mostly average) | No time unit | (Not reported/ European standard population) | Five | CI for incidence trends | Not reported | SPSS |
| Hassan 2016 | Not reported | Not reported | CRC cases/B6 | C5 | Not clear (mostly average) | No time unit | (Direct/ New WHO world standard population)* | Thirteen | Not reported | Not reported | SPSS |
| Abualkhair 2020 | ICD-O-3: site codes for primary CRC provided. | A1.1 | CRC cases | Not reported | Average | No time unit | (Not reported/ 2000 US standard population) | Thirty | CI for incidence rate and trends | Not reported | SEER |
| Al Dahhan 2018 | ICD-O-3 | Not reported | CRC cases | C5 | Not clear (mostly annual) | No time unit | Not applicable | Not reported | Not reported | Not reported | Not reported |
| Araghi 2018 | CRC site codes provided. | Not reported | CRC cases | Not reported | Not clear (mostly annual + average) | No time unit | (Not reported/ 2000 US standard population) | Twelve | Not reported | Not reported | Not reported |
| Araghi 2019 | ICD-10: site codes for primary CRC provided. | Not reported | CRC cases | Not reported | Not clear (mostly annual + average) | Person-time | (Not reported/ WHO world standard population) | Three | Not reported | Not reported | Not reported |
| Ashktorab 2016 | ICD-O-3: site description without codes. | Not reported | CRC cases | Not reported | Annual+ Average | No time unit | (Not reported/ 2000 US standard population) | One | Not reported | Not reported | SEER |
| Austin 2014 | ICD-O-3: site codes provided. | Not reported | CRC cases | Not reported | Annual | No time unit | (Not reported/ 2000 US standard million population) | Two | CI for incidence trends | Not reported | STATA |
| Aziz 2015 | ICD-O-3: morphological codes provided.  Conversion of codes not stated. | Not reported | CRC cases | Not reported | Not clear (mostly annual) | No time unit | Not applicable | Two/ Four | Not reported | Not reported | SAS |
| Bailey 2015 | Not reported | Not reported | CRC cases | Not reported | Annual | Whole years | (Not reported/ 2000 US standard population) | Four | CI for incidence trends | Not reported | SEER |
| Baniasadi 2015 | Not reported | Not reported | CRC cases | C3/C4 | Not clear (mostly annual) | Whole years | (Not reported/ WHO world standard population) | Seven | Not reported | Not reported | Microsoft Excel |
| Bhurgri 2011 | ICD-O-3: site codes provided. | A4/ A6 | CRC cases/B6 | C4/C5 | Average | Whole years | (Direct/ 1960 Segi’s world population) | Seven | Not reported | Not reported | SPSS |
| Boyce 2016 | ICD-10, Australian modification (AM) and ICD-O-3: site codes provided. | Not reported | CRC cases | Not reported | Not clear (mostly annual) | No time unit | (Not reported/ NSW population at 2001) | Two | CI for incidence trends | Not reported | STATA, SAS |
| Winther 2016 | ICD-10: site codes provided. Conversion of codes not stated. | Not reported | CRC cases | Not reported | Annual + Average | Person-time | Not applicable | Four | Not reported | Not reported | Not reported |
| Brenner 2016 | ICD-10: site codes provided. | Not reported | CRC cases | Not reported | Annual | No time unit | (Not reported/ European standard population) | Three | Not reported | Not reported | Not reported |
| Brouwer 2018 | ICD-O: site codes for primary CRC provided. | Not reported | CRC cases/B1 | Not reported | Annual | Person-time | (Not reported/ 1976 European standard population) | Not reported | CI for incidence trends | Not reported | STATA, SAS, SPSS |
| Caldarella 2013 | ICD-O-3: site codes provided. Conversion of codes not stated. | Not reported | CRC cases | Not reported | Average+ Annual (not clear) | Person-time | (Not reported/ 2000 European standard population) | Fourteen | CI for incidence trends | Not reported | Not reported |
| Carroll 2019 | ICD-10: site codes provided. Conversion of codes not stated | Not reported | CRC cases | C5 | Not clear (mostly annual) | Not reported | Not applicable | Not reported | Not reported | Not reported | Not reported |
| Chambers 2020 | ICD-9: site codes provided. ICD-10: site codes provided. | Not reported | CRC cases | C5 | Not clear (mostly annual) | Person-time | (Direct/ 2013 European standard population) | Four | CI for incidence trends | Not reported | Not reported |
| Chatterjee 2015 | Not reported | Not reported | CRC cases | C5 | Annual + Average | Whole years | (Not reported/ 2000 US standard population) | Four | Not reported | Not reported | Not reported |
| Alsanea 2015 | Not reported | Not reported | CRC cases | Not reported | Not clear (mostly annual) | No time unit | (Not reported/ Segi’s world population -modified by Doll) | Eight | Not reported | Not reported | Not reported |
| Chauvenet 2011 | ICD-O-3: site codes provided. Conversion of codes not stated. | A5 | CRC cases | C5 | Not clear (mostly average) | No time unit | (Direct/ WHO world standard population)* | Not reported | CI for incidence trends | Not reported | STATA |
| Chen 2012 | ICD-9- Clinical Modification (CM): site codes for primary and secondary CRC provided. | Not reported | CRC cases/B1 | C5 | Average | No time unit | Not applicable | Twenty/ Four | Not reported | Not reported | SPSS |
| Cheng 2011 | ICD-O-3: site codes for primary, in situ and invasive CRC provided. Conversion of codes not stated. | Not reported | CRC cases | Not reported | Not clear (mostly average) | Person-time | (Not reported/ 2000 US standard population) | Five | CI for incidence rate | Not reported | SEER |
| Chernyavskiy 2019 | Not reported | Not reported | CRC cases | Not reported | Average | Person-time | Not applicable | Two | CI for incidence rate and trends | Not reported | R |
| Chittleboroug-h 2020 | CRC sites are described without codes. | Not reported | CRC cases | Not reported | Annual + Average (not clear) | Whole years | (Not reported/ European standard population)* | Three | CI for incidence rate and trends | Not reported | R |
| Chong 2015 | Not reported | Not reported | CRC cases/B6 | C4/C5 | Not clear (mostly average) | No time unit | (Not reported/ WHO world standard population) | Fifteen | Not reported | Not reported | SPSS |
| Clarke 2014 | ICD-10: site codes for primary, invasive CRC provided. | A1.1 | CRC cases | Not reported | Not clear (mostly annual) | No time unit | (Direct/ European standard population) | Not reported | CI for incidence trends | Not reported | Not reported |
| Crocetti 2010 | Invasive CRC. | Not reported | CRC cases | Not reported | Not clear (mostly annual) | No time unit | (Not reported/ European standard population) | Two | CI for incidence trends | Not reported | Not reported |
| Crosbie 2018 | ICD-O-3: site codes for primary CRC provided. Excluded specific morphological codes. Conversion of codes not stated. | Not reported | CRC cases | Not reported | Annual (reported as annual rates in methods, but average rates were presented in results) | No time unit | (Not reported/ 2000 US standard population) | Three | CI for incidence rate and trends | D1/D5 | SEER |
| Danos 2018 | ICD-O-3: site codes for invasive primary CRC provided. Excluded specific morphological codes. | Not reported | CRC cases/B2/B3 | C1.2/C4/C5 | Average | No time unit | Not applicable | Four | Not reported | Not reported | SAS |
| Dehghani 2019 | ICD-O-2: site codes provided. | Not reported | CRC cases | C3/C4 | Not clear (mostly annual) | Person-time | Not applicable | Seven | Not reported | Not reported | Not reported |
| Edwards 2010 | ICD-O-3: site codes for first primary CRC provided. Conversion of codes not stated. | A6 | CRC cases/B5 | C5 | Annual + Average | No time unit | (Not reported/ 2000 US standard population) | Three | Not reported | Not reported | SEER |
| Ellis 2018 | Site codes provided for invasive and in situ CRC. | Not reported | CRC cases | C3/C4 | Annual + Average | No time unit | (Not reported/ 2000 US standard population) | Three | CI for incidence rate and trends | Not reported | SEER |
| Enayatrad 2018 | ICD-O-2: site codes provided. | Not reported | CRC cases | Not reported | 2009 | No time unit | (Direct/ WHO world standard population) | Not reported | Not reported | Not reported | SPSS |
| Eser 2018 | ICD-O-3: site codes for invasive CRC provided. | Not reported | CRC cases | Not reported | Not clear (mostly annual + average) | No time unit | (Direct/New WHO world standard population) | Not reported | CI for incidence rate | Not reported | SAS |
| Exarchakou 2019 | ICD-8/9/10: site codes for first primary, invasive CRC provided. | A1.1 | CRC cases | Not reported | Annual | No time unit | (Not reported/ European standard population) | Eight | CI for incidence trends | Not reported | Not reported |
| Feletto 2019 | CRC site codes provided. | Not reported | CRC cases | C5 | Annual | No time unit | Not applicable | Eleven | CI for incidence trends | Not reported | Not reported |
| Hasanpour-Heidari 2019 | ICD-O-3. Primary CRC. | A2 | CRC cases | C5 | Not clear (mostly annual + average) | Person-time | (Direct/ 1960 Segi’s world population) | Not reported | CI for incidence trends | Not reported | CanReg-5 |
| Lemmens 2010 | Site codes for primary CRC provided. | A1.1 | CRC cases/B2 | Not reported | Average | Person-time | (Not reported/ European standard population) | Not reported | Not reported | Not reported | Not reported |
| May 2017 | ICD-O-3. Conversion of codes not stated. | Not reported | CRC cases/B6 | Not reported | Not clear (mostly annual + average) | No time unit | (Not reported/ 2000 US standard population) | Not reported | Not reported | Not reported | SEER |
| Fusco 2010 | ICD-O-3: invasive CRC. | Not reported | CRC cases/B1/B3 | Not reported | Not clear (mostly average) | Whole years | (Not reported/ World, European and Italian standard populations) | Eight | Not reported | Not reported | STATA |
| Klugarova 2019 | ICD-10-Clinical Modification (CM). Conversion of codes not stated. | Not reported | CRC cases | Not reported | Not clear (mostly annual + average) | No time unit | Not applicable | Eighteen | Not reported | Not reported | Not reported |
| Koblinski 2018 | CRC sites are described without codes. | Not reported | CRC cases | C5 | Not clear (mostly annual) | No time unit | Not applicable | Two | Not reported | Not reported | SPSS |
| Martinsen 2016 | ICD-O-3: site codes for invasive CRC provided. Excluded specific morphological codes. | Not reported | CRC cases/B3/B6 | Not reported | Not clear (mostly annual) | No time unit | (Not reported/ 2000 US standard population) | Four | Not reported | Not reported | SEER |
| Giddings 2012 | ICD-O-3: site codes provided. Excluded specific morphological codes. Conversion of codes not stated. | A6 | CRC cases | C3/C4/C5 | Average + Annual (not clear) | No time unit | (Not reported/ 2000 US standard population) | Two | CI for incidence trends | Not reported | SEER |
| Missaoui 2011 | ICD-10: site codes provided. | Not reported | CRC cases | C5 | Not clear (mostly annual + average) | Person-time | (Direct/ New WHO world standard population) | Fourteen | CI for incidence trends | Not reported | Not reported |
| Kelly 2012 | ICD-O-2 &3: site codes provided. | Not reported | CRC cases | C5 | Average | No time unit | (Direct/ 2000 US standard population) | Seven | Not reported | Not reported | Not reported |
| Loomans-Kropp 2019 | ICD-O-3: site codes provided. Conversion of codes not stated. | Not reported | CRC cases | Not reported | Not clear (mostly annual) | No time unit | (Not reported/ 2000 US standard population) | Nine | CI for incidence trends | Not reported | SEER |
| Gandhi 2017 | CRC sites are described without codes. | Not reported | CRC cases | Not reported | Not clear (mostly annual) | No time unit | (Not reported/ 1976 European standard population) | Three | CI for incidence rate and trends | Not reported | Not reported |
| Lopez 2019 | ICD-O-3: site codes for invasive CRC provided. Conversion of codes not stated. | A5 | CRC cases/B1/B4 | C5 | Not clear (mostly average) | No time unit | (Direct/ WHO world standard population) | Three | Not reported | Not reported | Not reported |
| Lopez-Abente 2010 | ICD-9: site codes provided. ICD-10: site codes provided. | Not reported | CRC cases | C5 | Not clear (mostly average) | No time unit | (Direct/ European standard population) | Not reported | CI for incidence trends | Not reported | Not reported |
| McClements 2012 | ICD-10: site codes provided. Conversion of codes not stated. | Not reported | CRC cases | C5 | Not clear (mostly annual) | No time unit | Not applicable | One | Not reported | Not reported | SPSS, STATA |
| Gan 2019 | ICD-9 & 10. First primary, invasive CRC. | A5 | CRC cases/B5 | Not reported | Not clear | Not reported | Not applicable | Four | Not reported | Not reported | SAS |
| Ladabaum 2014 | ICD-O-3: site codes for primary invasive CRC provided. Excluded specific  morphological codes. | Not reported | CRC cases | C3/C4/C5 | Not clear (mostly annual + average) | No time unit | (Not reported/ 2000 US standard population) | Not reported | CI for incidence rate and trends | Not reported | SEER |
| Fowler 2018 | ICD-O-3 | Not reported | CRC cases | C5 | Annual + Average (not clear) | Person-time | (Not reported/ 2000 US standard population) | Not reported | CI for incidence rate | Not reported | SAS |
| Meester 2019 | Not reported | Not reported | CRC cases | Not reported | Not clear (mostly annual) | No time unit | (Not reported/ 2000 US standard population) | One | CI for incidence trends | Not reported | Joinpoint |
| Li 2017 | ICD-10: CRC sites are described without codes. | A6 | CRC cases | Not reported | Annual | Person-time | (Not reported/ 1960 Segi’s world population) | Four | CI for incidence trends | Not reported | Not reported |
| Liu 2015 | ICD-10: site codes for invasive CRC provided. | A2 | CRC cases | C3/C4/C5 | 2011 | No time unit | (Not reported/2000 Chinese population and 1960 Segi’s world population) | Eighteen | Not reported | Not reported | SAS |
| Jayarajah 2020 | ICD-10: site codes provided. | Not reported | CRC cases | Not reported | Annual | No time unit | (Not reported/ New WHO world standard population) * | Ten | CI for incidence rate and trends | Not reported | Not reported |
| Katsidzira 2016 | ICD-O-3: site and morphological codes provided. | A4 | CRC cases | C3/C4/C5 | Annual | No time unit | (Not reported/ Not reported) | Thirteen | CI for incidence trends | Not reported | STATA, CanReg- 4 |
| Fournel 2016 | First primary, invasive CRC. | A5 | CRC cases/B4/B7 | Not reported | Not clear (mostly average) | No time unit | (Direct/ New WHO world standard population) | Not reported | CI for incidence rate | Not reported | Not reported |
| Lee 2019 | ICD-10: site codes provided. Conversion of codes not stated. | Not reported | CRC cases | Not reported | Not clear (mostly average) | Person-time | Not applicable | Eleven | CI for incidence trends | Not reported | SAS, WinBUGS |
| Khiari 2017 | ICD-O-1 & 3: site codes provided. | A4 | CRC cases | C5 | Not clear (mostly average) | No time unit | (Direct/ Not reported) | Nine | Not reported | Not reported | SPSS |
| Shadmani 2017 | ICD-O | Not reported | CRC cases | Not reported | 2008 | Person-time | (Not reported/ Segi’s world population -modified by Doll) | Eighteen | Not reported | Not reported | Microsoft Excel |
| Merrill 2011 | ICD-O-2: site codes for primary and secondary malignant CRC provided. | Not reported | CRC cases | C5 | Not clear (mostly average) | No time unit | (Not reported/ 2000 US standard population) | Six | Not reported | Not reported | SEER, DevCan |
| Klimczak 2011 | Not reported | Not reported | CRC cases | Not reported | Not clear (mostly annual) | No time unit | (Not reported/ WHO world standard population) | Not reported | Not reported | Not reported | Not reported |
| Khiari; Ben Ayoube 2017 | ICD-O-1: site codes provided. ICD-O-3: site codes provided. | A4 | CRC cases | C5 | Not clear (mostly annual) | No time unit | (Not reported/ WHO world standard population) | Not reported | CI for incidence rate and trends | Not reported | R |
| Jandova 2016 | ICD-O-3: Morphological codes provided. | Not reported | CRC cases | Not reported | Not clear (mostly annual) | No time unit | Not applicable | Not reported | Not reported | D1/D5 | SPSS |
| Li; Lin 2017 | ICD-10: site codes provided. | Not reported | CRC cases | C4/C5 | Not clear (mostly annual) | No time unit | (Not reported/ 1964 Segi’s world population) | Not reported | CI for incidence trends | Not reported | Microsoft Excel |
| Meza 2010 | ICD-O-3: site codes provided. Conversion of codes not stated. | Not reported | CRC cases | C5 | Not clear (mostly annual) | No time unit | (Not reported/ 2000 US standard population) | Eighteen | Not reported | Not reported | Not reported |
| Jafri 2013 | ICD-O: site and morphological codes provided. | A1.2 | CRC cases | Not reported | Annual (reported as annual, but average rates were presented) | Person-time | (Not reported/ 2000 US standard population) | Six | CI for incidence rate | Not reported | SAS |
| McDevitt 2017 | ICD-O-3: site codes for primary, invasive CRC provided. | A1.1 | CRC cases | Not reported | Not clear (mostly average) | No time unit | (Not reported/ 1976 European standard population) | Not reported | CI for incidence trends | D2/D5/D6 | Not reported |
| Khachfe 2019 | ICD-O-3. Primary CRC. | Not reported | CRC cases | Not reported | Annual + Average | No time unit | (Not reported/ Ferlay’s modified world population) | Sixteen | CI for incidence trends | Not reported | Not reported |
| Meyer 2010 | CRC sites are described without codes. | Not reported | CRC cases | Not reported | Not clear (mostly annual + average) | No time unit | (Not reported/ 2000 US standard population) | One | CI for incidence rate and trends | Not reported | SEER |
| Garcia 2018 | ICD-O-3: CRC sites are described without codes. | Not reported | CRC cases | Not reported | Not clear (mostly annual + average) | No time unit | (Not reported/ 2000 US standard population) | Six | CI for incidence rate | D1/D5 | SEER |
| Fournel 2012 | ICD-O-2: morphological codes for first primary, invasive CRC provided. | A5 | CRC cases/B4 | C3/C4 | Not clear (mostly average) | Person-time | (Direct / New WHO world standard population) | Not reported | CI for incidence rate | Not reported | STATA |
| Brenner 2017 | ICD-O-3: site codes provided. Conversion of codes not stated. | Not reported | CRC cases | Not reported | Not clear (mostly annual) | No time unit | Not applicable | Eleven | Not reported | Not reported | Joinpoint, Age-Period-Cohort web tool (NCI) |
| Brenner 2019 | ICD-10: site codes provided. Conversion of codes not stated. | Not reported | CRC cases | Not reported | Not clear (mostly annual) | No time unit | (Not reported/Not reported) | Two | Not reported | Not reported | Joinpoint, Age-Period-Cohort web tool (NCI) |
| Fedewa 2019 | Not reported | Not reported | CRC cases | Not reported | Annual | No time unit | Not applicable | Three | CI for incidence rate and trends | Not reported | SEER |
| Augustus 2018 | ICD-O-3 | Not reported | CRC cases | Not reported | Not clear (mostly annual) | No time unit | (Not reported/ 2000 US standard population) | Three | Not reported | Not reported | SEER |
| Davis 2011 | Not reported | Not reported | CRC cases | C5 | Annual | No time unit | Not applicable | Eighteen | Not reported | Not reported | SEER |
| Domati 2014 | ICD-10. Conversion of codes not stated. | Not reported | CRC cases | C4 | Not clear (mostly annual) | Person-time | Not applicable | One | CI for incidence trends | Not reported | Joinpoint |
| Koblinski 2019 | CRC sites are described without codes. | Not reported | CRC cases | C5 | Not clear (mostly annual) | No time unit | Not applicable | Two | Not reported | Not reported | SEER |
| Purim 2013 | Malignant CRC. | Not reported | CRC cases/B6 | Not reported | Not clear (mostly average) | No time unit | (Not reported/ 2000 US standard population) | Eight | Not reported | Not reported | Not reported |
| Vuik 2019 | ICD-O-3: site codes provided. | A2 | CRC cases | Not reported | Average + Annual (not clear) | No time unit | (Not reported/ Population numbers for each country (not clear)) | Three | Not reported | Not reported | Not reported |
| Shafqat 2015 | ICD-O-3: morphological codes for invasive CRC provided. | A4 | CRC cases/B2/B3/B5/B6 | Not reported | Not clear (mostly annual + average) | Person-time | (Not reported/ 2000 US standard population) | Not reported | CI for incidence rate and trends | Not reported | STATA |
| Safaee 2012 | ICD-O-3: site codes provided. | Not reported | CRC cases | C4/C5 | Not clear (mostly average) | No time unit | (Direct/ WHO world standard population) | Four | CI for incidence rate | D1/D5 | SPSS, OpenEpi |
| Siegel 2017 | ICD-O-3: site codes provided. | Not reported | CRC cases | Not reported | Average | No time unit | (Not reported/ 2000 US standard population) | Three | Not reported | Not reported | SEER |
| Singh 2018 | ICD-9- Clinical Modification (CM): site codes provided. ICD-10-CA: site codes provided. | A5 | CRC cases | C5 | Annual + Average (not clear) | No time unit | (Not reported/ 2001 Canadian population) | Eight | Not reported | Not reported | Not reported |
| Savijarvi 2019 | CRC site codes provided. | Not reported | CRC cases | C5 | Average | No time unit | (Not reported/ WHO world standard population) | One | CI for incidence trends | D1/D5 | Not reported |
| Rahman 2015 | Not reported | Not reported | CRC cases | C5 | Not clear (mostly annual + average) | No time unit | (Not reported/ 2000 US standard population) | Two | Not reported | D3 | SEER |
| Nfonsam 2015 | ICD-O-3: morphological codes provided.  Conversion of codes not stated. | A6 | CRC cases | C5 | Not clear (mostly annual) | No time unit | Not applicable | Not reported | Not reported | Not reported | SAS |
| Van Beck 2018 | Not reported | Not reported | CRC cases | Not reported | Not clear (mostly annual) | No time unit | (Not reported/ 2000 US standard population) | Two | CI for incidence trends | Not reported | Joinpoint |
| Sammour 2009 | Not reported | Not reported | Colon cancer cases | C1.3/C4 | Not clear (mostly average) | Whole years | (Direct/ New WHO world standard population) | Not reported | Not reported | Not reported | SPSS |
| Mosli 2012 | Not reported | Not reported | CRC cases | Not reported | Not clear (mostly annual + average) | No time unit | Not applicable | Eighteen | Not reported | Not reported | Microsoft Excel |
| Mosli 2012 | Not reported | Not reported | CRC cases | Not reported | Annual | No time unit | Not applicable | Two | Not reported | Not reported | Microsoft Excel |
| Russo 2019 | ICD-O-3: site codes provided. Excluded specific morphological codes. | Not reported | CRC cases | Not reported | Not clear (mostly annual) | No time unit | Not applicable | Seven | CI for incidence trends | Not reported | Not reported |
| Sheneman 2017 | ICD-O-3: site codes provided. | A5 | CRC cases | Not reported | Average + Annual (not reported) | Person-time | (Direct/ 2000 US standard population) | Two | Not reported | Not reported | Microsoft Excel |
| Oliphant 2011 | ICD-10: site codes provided. | Not reported | CRC cases | C5 | Average + Annual (not reported) | No time unit | (Direct/ 1976 European Standard Population) | Not reported | CI for incidence rate | Not reported | STATA |
| Perdue 2014 | ICD-O-3 | Not reported | CRC cases | C5 | Not clear (mostly average) | No time unit | (Direct/ 2000 US standard population) | Three | Not reported | Not reported | SEER |
| Murphy 2017 | ICD-O-3: CRC sites are described without codes. Conversion of codes not stated. | Not reported | CRC cases | Not reported | Not clear (mostly average) | No time unit | (Not reported/ 2000 US standard population) | Nine | Not reported | Not reported | SEER |
| Shah 2012 | ICD-10: site codes provided. Conversion of codes not stated. | Not reported | CRC cases | C1.1/C4 | Not clear (mostly average) | Person-time | (Direct/ New WHO world standard population) | Two | CI for incidence rate and trends | Not reported | SAS |
| Siegel 2020 | ICD-O-3: site codes provided. | Not reported | CRC cases | C4/C5 | Average + Annual (not reported) | No time unit | (Not reported/ 2000 US standard population) | Three | Not reported | Not reported | SEER |
| Murphy 2011 | ICD-O-3: site codes provided. | Not reported | CRC cases | Not reported | Not clear (mostly average) | Person-time | (Not reported/ 2000 US standard population) | Nine | Not reported | Not reported | STATA |
| Shin 2012 | ICD-10: site codes provided. | Not reported | CRC cases | Not reported | Not clear (mostly annual) | No time unit | (Not reported/ 2000 Korean population) | Six | CI for incidence trends | Not reported | STATA |
| Patel 2016 | ICD-O-3: site codes provided. Conversion of codes not stated. | Not reported | CRC cases | Not reported | Not clear (mostly annual) | No time unit | (Not reported/ 1991 Canadian population) | Three | CI for incidence trends | Not reported | Not reported |
| Pakzad 2016 | ICD-O | Not reported | CRC cases | Not reported | 2009 | No time unit | (Direct/ WHO world standard population) | Not reported | Not reported | Not reported | Microsoft Excel |
| Siegel; Fedewa 2017 | ICD-O-3: site codes provided. Conversion of codes not stated. | Not reported | CRC cases | Not reported | Not clear (mostly annual) | Person-time | (Not reported/ 2000 US standard population) | Eleven | CI for incidence trends | Not reported | SEER |
| Pescatore 2013 | Nor clear | Not reported | CRC cases | Not reported | Annual | No time unit | (Direct/ WHO world standard population) | Sevent-een | Not reported | Not reported | Not reported |
| Murphy 2018 | CRC sites are described without codes. | Not reported | CRC cases | Not reported | Average | No time unit | (Not reported/ 2000 US standard population) | Six | Not reported | Not reported | SEER |
| Siegel 2012 | ICD-O-3: site codes provided. | Not reported | CRC cases | Not reported | Not clear (mostly annual) | No time unit | (Not reported/ 2000 US standard population) | One | CI for incidence trends | Not reported | SEER |
| Siegel 2019 | ICD-O-3: site codes provided. | Not reported | CRC cases | Not reported | Not clear (mostly average) | Person-time | (Not reported/ 2000 US standard population) | One | CI for incidence rate and trends | Not reported | SEER |
| Sung 2019 | Colon and rectal cancers were classified according to medical records. | Not reported | CRC cases | Not reported | Not clear (mostly annual) | No time unit | (Not reported/ New WHO world standard population) | One | CI for incidence trends | Not reported | Not reported |
| Rafiemanesh 2016 | ICD-O-3: site and morphological codes provided. | Not reported | CRC cases | Not reported | Not clear (mostly annual) | No time unit | (Direct/ WHO world standard population) | Not reported | CI for incidence trends | Not reported | Not reported |
| Sierra 2016 | ICD-10: site codes provided. | A3 | CRC cases | Not reported | Not clear (mostly average) | Person-time | (Direct/1960 Segi’s world population) | Not reported | Not reported | Not reported | STATA |
| Oliveira 2016 | ICD-10: site codes provided. Conversion of codes not stated. | Not reported | CRC cases | C5 | Not clear (mostly annual) | No time unit | (Not reported/ 1960 Segi’s world population) | Not reported | Not reported | Not reported | R |
| Palmieri 2013 | Not reported | Not reported | CRC cases | Not reported | Not clear (mostly average) | Whole years | (Not reported/ European standard population) | Sevent-een | Not reported | Not reported | Not reported |
| Paquette 2015 | CRC sites are described without codes. | Not reported | CRC cases | Not reported | Not clear (mostly average) | No time unit | (Not reported/ 2000 US standard population) | Thirty-three | Not reported | Not reported | SEER |
| Reggiani-Bonetti 2013 | ICD-O | Not reported | CRC cases | C4 | Not clear (mostly average) | Whole years | (Not reported/ Italy, Europe, World standard population) | Not reported | CI for incidence trends | Not reported | Joinpoint |
| Nowicki 2018 | ICD-10: site codes for malignant CRC provided. | Not reported | CRC cases | Not reported | Not clear (mostly annual) | No time unit | Not applicable | Not reported | Not reported | Not reported | Statistica, Microsoft Excel |
| Phipps 2012 | ICD-O-3: site codes for invasive CRC provided. Conversion of codes not stated. | Not reported | CRC cases/B3 | Not reported | Annual | No time unit | (Not reported/ 2000 US standard population) | Three | CI for incidence trends | Not reported | Not reported |
| Oppelt 2019 | ICD-10 German modification (GM). | A6 | CRC cases | Not reported | Annual | No time unit | (Not reported/ 1976 European standard population) | Not reported | Not reported | Not reported | SAS |
| Murphy 2019 | CRC sites are described without codes. | A1.1 | CRC cases | Not reported | Not clear (mostly average) | No time unit | (Not reported/ 2000 US standard population) | One | Not reported | Not reported | SEER |
| Innos 2018 | ICD-10: site codes provided. | A2 | CRC cases | Not reported | Not clear (mostly annual) | No time unit | (Not reported/ WHO world standard population) | Four | CI for incidence trends | Not reported | Not reported |
| Siegel 2014 | ICD-O-3: site codes provided. Conversion of codes not stated. | Not reported | CRC cases | Not reported | Not clear (mostly average) | No time unit | (Not reported/ 2000 US standard population) | Three | Not reported | Not reported | SEER |
| Sia 2014 | ICD: site codes provided. | Not reported | CRC cases | C5 | Not clear (mostly annual) | No time unit | Not applicable | Two | CI for incidence trends | Not reported | Not reported |
| Rejali 2018 | ICD-O-3: site codes for first primary CRC provided. | Not reported | CRC cases | C5 | Annual | No time unit | (Direct/ 2000 US standard population) | Not reported | CI for incidence rate and trends | D4/D5/D6 | STATA |
| Sarakarn 2017 | ICD-O: site codes provided. | Not reported | CRC cases | Not reported | Annual | No time unit | (Direct/ Segi’s world population -modified by Doll) | Four | Not reported | Not reported | Not reported |
| Keum 2014 | Invasive cancers. | Not reported | CRC cases | Not reported | Not clear (mostly annual) | No time unit | (Not reported/ 2000 US standard population) | Three | Not reported | Not reported | SEER |
| Singh 2014 | ICD-O-3: morphological codes for malignant CRC provided. Conversion of codes not stated. | A7 | CRC cases | C5 | Average + Annual | No time unit | Not applicable | Seven | CI for incidence rate | Not reported | SAS |
| Sjostrom 2018 | ICD | Not reported | CRC cases | C5 | Not clear (mostly average) | Person-time | (Not reported/ 2000 Swedish population) | Four | Not reported | Not reported | R |
| Steinbrecher 2012 | ICD-O-2: site codes for first primary, invasive CRC provided. | Not reported | CRC cases | C1.2/C4/C5 | Not clear (mostly average) | No time unit | (Not reported/ 2000 US standard population) | One | CI for incidence rate | Not reported | SEER |
| Stern 2016 | ICD-O-3 | Not reported | CRC cases | C1.2/C4/C5 | Not clear (mostly average) | No time unit | (Not reported/ 2000 US standard population) | Not reported | CI for incidence rate | Not reported | Not reported |
| Stock 2012 | ICD-O-3: site codes provided.  Conversion of codes not stated. | Not reported | CRC cases | Not reported | Not clear (mostly average) | Not applicable | Not applicable | Five | CI for incidence rate | Not reported | Not reported |
| Stromberg 2019 | First primary CRC. | Not reported | CRC cases | C5 | Average | Whole years | Not applicable | Twelve | Not reported | Not reported | Rapid Inquiry Facility, R, SPSS |
| Sun 2020 | ICD-7: site codes for first primary CRC provided. | Not reported | CRC cases | Not reported | Not clear (mostly average) | Person-time | (Direct/ 2000 Swedish population) | Two | CI for incidence trends | Not reported | SAS |
| Tawadros 2015 | ICD-9: site codes provided. | Not reported | Rectal cancer cases | Not reported | Not clear (mostly annual) | No time unit | Not applicable | Two | Not reported | Not reported | SEER |
| Thirunavu-karasu 2010 | ICD-O-3: morphological code for malignant CRC provided. Conversion of codes not stated. | Not reported | CRC cases | Not reported | Average + Annual (not clear) | No time unit | Not applicable | Five | CI for incidence rate | Not reported | SPSS |
| Thuraisingam 2017 | Not reported | Not reported | Colon cancer cases | Not reported | Not clear (mostly annual) | No time unit | Not applicable | Not reported | Not reported | Not reported | SPSS |
| Troeung 2017 | ICD-9-Clinical Modifications (CM): site codes for first primary CRC provided. ICD-10- Australian Modification (AM): site codes for first primary CRC provided. | Not reported | CRC cases | C5 | Average | No time unit | (Direct/ 2001 Australian population) | Five | CI for incidence trends | Not reported | Not reported |
| Ugarte 2012 | ICD-10: site codes provided. Conversion of codes not stated. | Not reported | CRC cases | C5 | Not clear (mostly annual + average) | No time unit | (Not reported/ European standard population) | Three | Not reported | Not reported | R |
| Ullah 2018 | Not reported | A1.1 | CRC cases/B6 | C5 | Not clear (mostly annual) | No time unit | (Not reported/ 1976 European standard population) | Seven | Not reported | Not reported | Not reported |
| Veruttipong 2012 | Not reported | A5 | CRC cases | C5 | Not clear (mostly average) | No time unit | (Not reported/ Not reported) | Nineteen | CI for incidence rate | Not reported | SAS |
| Wan Ibrahim 2020 | Not reported | Not reported | CRC cases | C5 | Not clear (mostly annual + average) | No time unit | (Not reported/ New WHO world standard population) | Two | Not reported | Not reported | R |
| Wang 2017 | ICD-O-3: site and morphological codes for primary, invasive CRC provided. | A6 | CRC cases | Not reported | Not clear (mostly annual + average) | No time unit | (Direct/ 2000 US standard population) | Four | CI for incidence trends | Not reported | SEER |
| Wang; de-Grubb 2017 | ICD-O-3: site codes provided | Not reported | CRC cases | Not reported | Annual + Average (not clear) | Person-time/ Whole years | (Not reported/ 2000 US Standard population) | Five | CI for incidence rate and trends | Not reported | SEER, SPSS |
| Wang 2019 | ICD-O-3: site codes for primary CRC provided. Conversion of codes not stated. | Not reported | CRC cases | Not reported | Annual | No time unit | (Not reported/ 2000 US standard population) | One | Not reported | Not reported | SAS |
| Wen 2018 | ICD-10: site codes provided. | A5 | CRC cases | C5 | Annual | No time unit | (Not reported/ Segi’s world population -modified by Doll) | Eighteen | Not reported | Not reported | Not reported |
| Wessler 2010 | ICD-10: site codes provided. Conversion of codes not stated. | A1.1/ A3 | CRC cases | C5 | Not clear (mostly average) | Person-time | (Direct/ European standard population) | Twelve | CI for incidence trends | Not reported | STATA |
| Wu 2018 | ICD-10: site codes provided. Conversion of codes stated. | Not reported | CRC cases | C2/C3/C5 | Not clear (mostly average) | No time unit | (Direct/ 1960 Segi’s world population) | Eleven | CI for incidence trends | Not reported | Not reported |
| Yee 2010 | ICD-10: site codes provided. Conversion of codes not stated. | A4 | CRC cases | C5 | Average + Annual (not clear) | No time unit | (Not reported/ WHO world standard population) | Six | Not reported | Not reported | Not reported |
| Yeo 2017 | First primary CRC. | Not reported | CRC cases | Not reported | Not clear (mostly annual) | No time unit | (Not reported/ 2000 US standard population) | Two | Not reported | Not reported | SEER, STATA |
| Yoon 2015 | ICD-10: site codes provided. | Not reported | CRC cases | C5 | Not clear (mostly annual) | No time unit | (Not reported/ 1960 Segi’s world population)* | Fourteen | Not reported | Not reported | Not reported |
| Young 2015 | ICD-O-3: site codes for primary, invasive CRC provided. | Not reported | CRC cases | C4/C5 | Not clear (mostly average) | No time unit | (Direct/ 1991 Canadian population) | Three | CI for incidence rate | Not reported | SEER |
| Zhabagin 2015 | First primary CRC. | Not reported | CRC cases | C5 | Average + Annual (not clear) | No time unit | Not applicable | Six | Not reported | Not reported | Not reported |
| Zhang 2018 | Not reported | Not reported | CRC cases | C5 | Annual + Average | Person-time | (Direct/ Segi’s world population -modified by Doll) | Three | CI for incidence trends | Not reported | Not reported |
| Zheng 2014 | ICD-10: site codes provided. | A2 | CRC cases | C5 | 2010 | No time unit | (Not reported/ 2000 Chinese standard population and 1960 Segi’s world population) | Fourteen | Not reported | Not reported | Microsoft FoxPro, Microsoft Excel, SAS |
| Zhou 2015 | ICD-10: site codes provided. | Not reported | CRC cases | C5 | Annual | No time unit | (Not reported/ 1960 Segi’s world population) | Three | Not reported | Not reported | SAS |
| Zhu 2013 | ICD-O-3: site codes for primary, in situ and invasive colon cancer provided. Conversion of codes not stated. | Not reported | colon cancer cases | Not reported | Not clear (mostly average) | No time unit | (Not reported/ 2000 US standard population) | Nine | Not reported | Not reported | SEER |
| Zorzi 2019 | ICD-10: site codes provided. | A1.2/A3 | CRC cases | Not reported | Not clear (mostly annual) | No time unit | (Not reported/ 2013 European standard population) | Three | CI for incidence trends | D2/D5 | Not reported |
| Zorzi 2015 | ICD-10: site codes for primary and secondary CRC provided. | Not reported | CRC cases/B1/B5 | Not reported | Annual | No time unit | (Not reported/ 2001 European population) | Six | CI for incidence trends | Not reported | Not reported |
| Ohri 2020 | Not reported | Not reported | CRC cases | Not reported | Annual + Average (not clear) | Whole years | (Not reported/ 2000 US standard population) | Ten | CI for incidence rate | Not reported | SEER |
| *Study justified the chosen standard population  **Abbreviations:** ICD: International classification of disease, ICD-0: International classification of disease for oncology, CRC: Colorectal cancer, CI: Confidence interval, NCI: National cancer institute. | | | | | | | | | | | |
| **Quality assessment of registry data.** A1: Study cited a reference for previously conducted research as evidence of cancer registry data quality: A1.1: Study referenced other studies or reports including validation or completeness assessments, A1.2: Study referenced similar epidemiological studies conducted in the same data source; A2: Study assessed and reported certain validity indicators; A3: Study reported specific validity indicators from external resources; A4: Study reported that a cancer registration program checked data quality; A5: Study indicated that the registration quality is being audited and certified regularly by a certification body; A6: Study indicated that cancer registry is meeting or utilizing standards for data quality set by national or international agencies; A7: Study indicated complete case ascertainment of cancer data without providing a reference. | | | | | | | | | | | |
| **Definition of the numerator.** B1: Information provided about considerations for synchronous and metachronous CRC cases in incidence calculation; B2: Exclude cases with an unknown site of the primary tumour or disease stage or survival time; B3: Excluded In situ cancers; B4: Exclude cases with family history, hereditary syndromes, and IBD; B5: Exclude cases identified by only death certificate; B6: Exclude non-microscopically confirmed cases; B7: Exclude cases with incomplete address information. | | | | | | | | | | | |
| **Definition of the denominator.** C1: Explicit explanation of population size estimation: C1.1: The study calculated person-time at risk by creating closed cohorts of the population on various census nights and following them over time, C1.2: Population size was estimated by multiplying the population count in a particular census year by the number of years included in the study, C1.3: The denominator size was calculated by averaging population counts of two censuses conducted at the beginning and near the end of the study period. C2: The annual mid-year population is estimated by averaging the populations at the end of the adjacent years. C3: Yearly population counts are interpolated and extrapolated. C4: Reporting of census years that were used for population size estimation. C5: Data source of the general population is reported. | | | | | | | | | | | |
| **Assessment of missing data.** D1: Study excluded cases with missing values from incidence calculation; D2: Study estimated missing data; D3: Study assumed missing data to be missing at random; D4: Study corrected rates for missing data; D5: Study reported type of missing data; D6: Study indicated the amount of missing data. | | | | | | | | | | | |
