# Supplementary material for: A systematic review of methods to estimate colorectal cancer incidence using population-based cancer registries
Source: BMC Med Res Methodol. 2022 May 19;22:144. doi: 10.1186/s12874-022-01632-7 (PMC9118801; doi:10.1186/s12874-022-01632-7)
Supplement: Supplementary file 3 — Additional file 3. Results tables. [file 12874_2022_1632_MOESM3_ESM.docx]

**Additional File 3** Results tables (P.1-8)

| **Table 3.1** Title/Abstract screening inter-reviewer agreement rate calculation (κ statistic) | | | | |
| --- | --- | --- | --- | --- |
|  | **Reviewer (AA)** | | | |
|  |  | Included | Excluded | Total |
| **Reviewer (NA)** | Included | 234 (a) | 5 (b) | 239 |
|  | Excluded | 15 (c) | 2652(d) | 2667 |
|  | Total | 249 | 2657 | 2906 |

$$p_{0}=\frac{\left( a+d \right)}{a+d+b+c}=\frac{\left( 234+2649 \right)}{2906}=99\%$$

$$p_{e}=\left[ \left( \frac{a+b}{n} \right)* \left( \frac{a+c}{n} \right) \right]+\left[ \left( \frac{c+d}{n} \right)*\left( \frac{b+d}{n} \right) \right]=$$

$$\left[ \left( \frac{239}{2906} \right)* \left( \frac{249}{2906} \right) \right]+\left[ \left( \frac{2667}{2906} \right)*\left( \frac{2657}{2906} \right) \right]=84\%$$

$$\kappa=\frac{\left( p_{0}-p_{e} \right)}{\left( 1-p_{e} \right)}=\frac{\left( 99\%-84\% \right)}{\left( 1-84\% \right)}=94\%$$

| **Table 3.2** Description of characteristics of included studies | |
| --- | --- |
| **Study characteristics** | |
| ***Country*** | ***N (% out of 165)*** |
| The United States of America | 66 (40.0) |
| Europe | 38 (23.0) |
| Asia | 36 (21.8) |
| Oceania | 7 (4.2) |
| Canada | 5 (3.0) |
| Africa | 5 (3.0) |
| Multi-country | 6 (3.6) |
| Central and South America | 2 (1.2) |
| ***Main outcomes*** *(presented here the three most common outcomes reported in the included studies)* | ***N (% out of 165)*** |
| Incidence | 165 (100.0) |
| Mortality | 41 (24.8) |
| Survival | 36 (21.8) |
| ***Type of cancer*** | ***N (% out of 165)*** |
| Colorectal cancer (CRC) | 160 (96.9) |
| Colon cancer | 3 (1.8) |
| Rectum cancer | 2 (1.2) |
| ***Observation period*** | ***N (% out of 165)*** |
| A single year | 5 (3.0) |
| Less than 10 years | 32 (19.4) |
| 10-19 years | 61 (36.9) |
| 20 years or more | 69 (41.8) |
| **Reported incidence rate measures** | |
| ***Type of measures used for reporting incidence*** (Some studies reported more than one incident measure) | ***N (% out of 165)*** |
| Age-standardized incidence rate (ASR) | 132 (80.0) |
| Age-specific incidence rate (ASIR) | 50 (30.3) |
| Crude incidence rate (CR) | 31 (18.8) |
| Cumulative incidence rate | 3 (1.8) |
| Cumulative risk | 7 (4.2) |
| Truncated ASR | 3 (1.8) |
| Delay adjusted rate | 4 (2.4) |
| Incidence rate   - Derived from modelling. - Reported as the frequency of new cases. - Reported as the percentage of CRC cases among various groups. | 18 (10.9)  4 (2.4)  2 (1.2)  2 (1.2) |
| Risk adjusted rate | 1 (0.6) |
| Only ASR | 74 (44.8) |
| Only ASIR | 9 (5.5) |
| Only CR | 4 (2.4) |
| Only ASR + ASIR | 28 (16.9) |
| Only ASR + CR | 14 (8.5) |
| Only ASR +ASIR + CR | 12 (7.3) |
| Only ASIR + CR | 1 (0.6) |
| ***The standard population used in estimating the ASR*** | ***N (% out of 127 studies that reported the standard population)*** |
| Study employed a local reference population | 64 (50.4) |
| Study employed an external reference population | 71 (55.9) |
| Study employed both a local and an external standard population | 4 (3.1) |
| ***Used standard population according to study aim*** | ***N (% out of studies with a specific aim (13,114))*** |
| Study conducted international comparisons (all used an external reference):   - Used same external reference population. - Used different external reference populations. | 13  10 (76.9)  3 (23.1) |
| Study assessed only local incidence rates and trends:   - Used a local reference. - Used an external reference. | 114  62 (54.4)  52 (45.6) |
| ***Stratification of CRC incidence rate by anatomical site*** | ***N (% out of 160 studies that reported CRC incidence)*** |
| According to CRC anatomical site | 86 (53.8) |
| Anatomical sites chosen for reporting CRC incidence: (some studies used a combination of the below sites) | ***N*** ***(% out of 86 studies that reported incidence stratified by anatomical site)*** |
| - Colon/Rectum. | 77 (89.5) |
| - Proximal/Distal colon. | 33 (38.4) |
| - Right-sided/Left-sided tumours. | 11 (12.8) |
| - Colon, not otherwise specified (NOS). | 7 (8.1) |
| - Appendix. | 6 (6.9) |
| - Overlapping lesion of the colon. | 2 (2.3) |
| - Rectosigmoid junction. | 6 (6.9) |
| - Anus. | 4 (4.7) |
| - Multiple anatomical sites in the colon. | 7 (8.1) |
| Study reported only site-specific incidence rate without total CRC rate | 29 (33.7% out of 86) |
| **Quality of reporting incidence** | |
| ***Quality of cancer registry data*** | ***N (% out of 165)*** |
| Study cited a reference for previously conducted research as evidence of cancer registry data quality:   - Study referenced other studies or reports including validation or completeness assessments. - Study referenced similar epidemiological studies conducted in the same data source. | 10 (6.1)  8 (4.8)  2 (1.2) |
| Study assessed and reported specific validity indicators | 5 (3.0) |
| Study reported specific validity indicators that were identified in an external reference | 3 (1.8) |
| Study reported that a cancer registration program checked data quality | 6 (3.6) |
| Study indicated that the registration quality is being audited and certified regularly by a certification body | 9 (5.5) |
| Study indicated that the cancer registry is meeting or utilizing standards for data quality set by national or international agencies | 7 (4.2) |
| Study indicated complete case ascertainment of cancer data without providing a reference study | 1 (0.6) |
| ***Definition of colorectal cancer*** | ***N (% out of the indicated number)*** |
| **Classification system** | |
| Study reported the use of a classification system | 120 (72.7% out of 165) |
| - ICD-O | 78 (65.0% out of 120) |
| - 1^st^ edition | 2 (1.7% out of 120) |
| - 2^nd^ edition | 7 (5.8% out of 120) |
| - 3^rd^ edition | 63 (52.5% out of 120) |
| - No edition specified | 6 (5.0% out of 120) |
| - ICD | 53 (44.2% out of 120) |
| - 7^th^ edition | 1 (0.8% out of 120) |
| - 8^th^ edition | 1 (0.8% out of 120) |
| - 9^th^ edition | 8 (6.7% out of 120) |
| - 10^th^ edition | 40 (33.3% out of 120) |
| - No edition specified | 3 (2.5% out of 120) |
| Study reported a classification system but without describing CRC codes | 23 (13.9% out of 165) |
| Study did not report a classification system: | 45 (27.3% out of 165) |
| - No reporting of the classification system and CRC codes. | 32 (19.4% out of 165) |
| - No reporting of classification system but providing CRC site codes. | 5 (3.0% out of 165) |
| - No reporting of classification system but describing only CRC sites. | 8 (4.8% out of 165) |
| **CRC codes** | |
| Study did not report CRC codes | 63 (38.2% out of 165) |
| Study reported CRC codes | 102 (61.8% out of 165) |
| Study reported morphology codes only | 6 (3.6% out of 165) |
| Study reported topography codes only | 85 (51.5% out of 165) |
| Study reported morphology and topography codes | 11 (6.7% out of 165) |
| **Tumour behaviour** |  |
| Study explicitly indicated tumour behaviour (malignant/ in situ, other) | 28 (16.9% out of 165) |
| **Type of cancer** |  |
| Study specified the included cancer type (primary/secondary) | 31 (18.8% out of 165) |
| ***Definition of numerator and denominator data*** | ***N (% out of 165)*** |
| **Numerator** |  |
| Study explicitly explained excluding certain CRC cases from the numerator | 20 (12.1) |
| Study excluded cases with unknown sites of primary tumours, or disease stage, or survival time | 3 (1.8) |
| Study excluded in situ cancers | 5 (3.0) |
| Study excluded cases with family history, hereditary syndromes, and IBD | 3 (1.8) |
| Study excluded cases identified by only death certificate | 4 (2.4) |
| Study excluded non-microscopically confirmed cases | 8 (4.8) |
| Study excluded cases with incomplete address information | 1 (0.6) |
| Study provided information about considerations for secondary CRC (synchronous and metachronous cancer cases) in incidence calculation | 5 (3.0) |
| **Denominator** |  |
| Study reported the data source for population statistics | 65 (39.4) |
| Study reported the estimation of annual mid-year population | 1 (0.6) |
| Study reported census years used for population size estimation | 24 (14.5) |
| Study reported the method used to estimate yearly population counts (i.e., interpolation, extrapolation) | 10 (6.1) |
| Study explicitly explained the population size estimation procedure for calculating average incidence rates (over the study period): | 5 (3.0) |
| - Person-time at risk was calculated by creating closed cohorts of the population on various census nights and following them over time. | 1 (0.6) |
| - Population size was estimated by multiplying the population count in a particular census year by the number of years included in the study. | 3 (1.8) |
| - Population size was calculated by averaging population counts of two censuses conducted at the beginning and near the end of the study period. | 1 (0.6) |
| ***Estimation of the age-standardized rate*** | ***N (% out of 132)*** |
| Study reported the method of standardization: | 36 (27.3) |
| - Direct. | 36 (27.3) |
| - Indirect. | 0 |
| Study reported the standard population used for standardization | 127 (96.2) |
| Study did not report the standard population used for standardization | 5 (3.8) |
| Study justified the chosen standard population | 5 (3.9% out of 127) |
| ***Time interval for measuring incidence (e.g., annual, overall average)*** | ***N (% out of 165)*** |
| Time interval was clearly reported | 62 (37.6) |
| Time interval was not clearly reported | 103 (62.4) |
| Study reported an overall incidence measure for a specific observation period | 90 (54.5) |
| ***Presentation of incidence rates*** | ***N (% out of 165)*** |
| Incidence rate was expressed with a time unit (whole years or person-time) | 43 (26.1) |
| Incidence rate was expressed without a time unit | 119 (72.1) |
| ***Age band for measuring incidence*** | ***N (% out of 165)*** |
| Age bands were clearly reported. | 131 (79.4) |
| *Number of age bands* |  |
| - - 1 | 12 (7.3) |
| - - 2 | 19 (11.5) |
| - - 3 | 23 (13.9) |
| - - 4 | 15 (9.1) |
| - - 5-10 age bands | 35 (21.2) |
| - - 11-15 age bands | 13 (7.9) |
| - - 16-20 age bands | 12 (7.3) |
| - - 30-33 | 2 (1.2) |
| ***Estimation of uncertainty of incidence estimates*** | ***N (% out of 165)*** |
| Study reported a confidence interval for the incidence rate | 33 (20.0) |
| ***Analysis of missing data*** | ***N (% out of 165)*** |
| Study reported handling of missing data in the analysis: | 9 (5.5) |
| - Study excluded cases with missing values from incidence calculation. | 5 (3.0) |
| - Study estimated missing data via join point regression or multiple imputation. | 2 (1.2) |
| - Study assumed missing data to be missing at random. | 1 (0.6) |
| - Study corrected rates for missing data. | 1 (0.6) |
| Study reported the type of missing data | 8 (4.8) |
| Study indicated the amount of missing data | 2 (1.2) |
| Study justified assumption on the reasons for the missing data | 0 |
| ***Statistical software*** | ***N (% out of 165)*** |
| Study reported software information for incidence rate analysis | 110 (66.7) |
| Software reported for estimating incidence rate: | ***N (% out of 110)*** |
| - SEER | 40 (36.4) |
| - SAS | 19 (17.3) |
| - STATA | 18 (16.4) |
| - SPSS | 17 (15.5) |
| - Microsoft Excel | 9 (8.2) |
| - R | 8 (7.3) |
| - Can Reg4/CanReg-5 | 2 (1.8) |
| - Joinpoint | 6 (5.5) |
| - Microsoft Fox Pro | 1 (0.9) |
| - WinBUGS | 1 (0.9) |
| - Open-Source Epidemiologic Statistics for Public Health software (OpenEpi) | 1 (0.9) |
| - Statistica | 1 (0.9) |
| - Rapid Inquiry Facility | 1 (0.9) |
| - DevCan | 1 (0.9) |

| **Table 3.3.** Types of standard populations employed for the calculation of age-standardized rates (ASR) | | | |
| --- | --- | --- | --- |
|  | **Reference Population** | **N** **(% out of 132 studies reported ASR)** | **Countries** |
| 1 | 2000 US standard population | 52 (39.4) | United States of America, United Kingdom |
| 2 | WHO world standard population | 27 (20.5) | Iran, Malaysia, seven high-income countries, France, Brunei Darussalam, Cyprus, Jordan, Israel, Izmir, Turkey, Italy, Tunisia, Sri- Lanka, Poland, Finland, New- Zealand, Luxembourg, Hong Kong, Korea, Japan, Taiwan, Estonia |
| 3 | Segi's world population | 11 (8.3) | Pakistan, Iran, China, Central and South America, Shanghai, Korea |
| 4 | Segi’s standard world population (modified by Doll) | 5 (3.8) | China, Hong Kong, Thailand, Iran, Saudi Arabia |
| 5 | Ferlay's modified world population | 1 (0.8) | Lebanon |
| 6 | European standard population | 13 (9.8) | Portugal, New Zealand, United Kingdom, Ireland, Germany Sweden, Scotland, Italy, Spain, Netherlands |
| 7 | The 2001 European standard population | 1 (0.8) | Italy |
| 8 | The 2000 European standard population | 1 (0.8) | Italy |
| 9 | The 2013 European standard Population | 2 (1.5) | United Kingdom, Italy |
| 10 | The 1976 European standard population | 6 (4.5) | New Zealand, Ireland, United Kingdom, Netherlands, Germany, Ireland |
| 12 | The 2000 Chinese population | 2 (1.5) | China |
| 13 | The Italian populations | 2 (1.5) | Italy |
| 14 | The 2001 Canadian population | 1 (0.8) | Canada |
| 15 | The 1991 Canadian population | 2 (1.5) | Canada |
| 16 | The 2000 Swedish population | 2 (1.5) | Sweden |
| 17 | The 2001 Australian population | 1 (0.8) | Australian |
| 18 | The 2000 Korean population | 1 (0.8) | Korea |
| 19 | The 2001 New South Wales (NSW) population | 1 (0.8) | Wales |
