# Supplementary material for: A systematic review of methods to estimate colorectal cancer incidence using population-based cancer registries
Source: BMC Med Res Methodol. 2022 May 19;22:144. doi: 10.1186/s12874-022-01632-7 (PMC9118801; doi:10.1186/s12874-022-01632-7)
Supplement: Supplementary file 4 — Additional file 4. Quality assessment. [file 12874_2022_1632_MOESM4_ESM.docx]

**Additional file 4** Quality assessment (P.1-7)

|  | | | |
| --- | --- | --- | --- |
| Table 4.1 Quality appraisal checklist | | | |
| Item | **Yes** | **No** | **Unclear** |
| 1. Were the aims/objectives of the study clear? * |  | |  |
| 1. Was the study design appropriate for the stated aim(s)? * |  | |  |
| 1. Was the sample size adequate? § |  | |  |
| 1. Were the study subjects and setting described in detail? § |  | | |
| 1. Were valid methods used for the identification of the condition? § |  | | |
| 1. Were the risk factor and outcome variables measured correctly using instruments/measurements that had been trialed, piloted, or published previously?* |  | | |
| 1. Was there an appropriate statistical analysis? § |  | | |
| 1. Is it clear what was used to determine statistical significance and/or precision estimates? (e.g., p values, CIs)? * |  | | |
| 1. Were the methods (including statistical methods) sufficiently described to enable them to be repeated? * |  | | |
| 1. Were the limitations of the study discussed? * |  | | |

**§** Item from the Joanna Briggs Institute Prevalence Critical Appraisal Tool

*Item from the AXIS tool

| **Table 4.2** Quality assessment. Studies are sorted in order from highest to lowest quality | | | | | | | | | | | |
| --- | --- | --- | --- | --- | --- | --- | --- | --- | --- | --- | --- |
| **First author and year** | **Clear aim and objectives** | **Appropriate study design** | **Adequate sample size** | **Description of study subjects and setting** | **Valid methods to identify the condition** | **Outcome variables were measured correctly using instruments/measurements that had been published previously** | **Appropriate statistical analysis** | **Determine precision estimates** | **Methods sufficiently described to enable them to be repeated** | **limitations of the study discussed** | **Total** |
| Steinbrecher 2012 | √ | √ | √ | √ | √ | √ | √ | √ | √ | √ | 10 |
| Danos 2018 | √ | √ | √ | √ | √ | √ | √ | X | √ | √ | 9 |
| Stern 2016 | √ | √ | √ | √ | X | √ | √ | √ | √ | √ | 9 |
| Crosbie 2018 | √ | √ | √ | √ | √ | √ | X | √ | X | √ | 8 |
| Jayarajah 2020 | √ | √ | √ | √ | √ | √ | X | √ | X | √ | 8 |
| Katsidzira 2016 | √ | √ | √ | √ | √ | √ | √ | X | X | √ | 8 |
| Shafqat 2015 | √ | √ | √ | √ | √ | √ | X | √ | X | √ | 8 |
| Safaee 2012 | √ | √ | √ | √ | √ | √ | X | √ | X | √ | 8 |
| Shah 2012 | √ | √ | √ | √ | √ | √ | √ | X | X | √ | 8 |
| Patel 2016 | √ | √ | √ | √ | √ | √ | X | √ | X | √ | 8 |
| Siegel 2019 | √ | √ | √ | √ | √ | √ | X | √ | X | √ | 8 |
| Wu 2018 | √ | √ | √ | √ | √ | √ | √ | X | X | √ | 8 |
| Young 2015 | √ | √ | √ | √ | √ | √ | X | √ | X | √ | 8 |
| Abdifard 2016 | √ | √ | √ | √ | √ | √ | √ | X | X | X | 7 |
| Abualkhair 2020 | √ | √ | √ | √ | √ | √ | X | X | X | √ | 7 |
| Araghi 2019 | √ | √ | √ | √ | √ | √ | X | X | X | √ | 7 |
| Austin 2014 | √ | √ | √ | √ | √ | √ | X | X | X | √ | 7 |
| Aziz 2015 | √ | √ | √ | √ | √ | √ | X | X | X | √ | 7 |
| Boyce 2016 | √ | √ | √ | √ | √ | √ | X | X | X | √ | 7 |
| Brenner 2016 | √ | √ | √ | √ | √ | √ | X | X | X | √ | 7 |
| Brouwer 2018 | √ | √ | √ | √ | √ | √ | X | X | X | √ | 7 |
| Caldarella 2013 | √ | √ | √ | √ | √ | √ | X | X | X | √ | 7 |
| Carroll 2019 | √ | √ | √ | √ | √ | √ | X | X | X | √ | 7 |
| Chambers 2020 | √ | √ | √ | √ | √ | √ | X | X | X | √ | 7 |
| Cheng 2011 | √ | √ | √ | √ | √ | √ | X | X | X | √ | 7 |
| Chernyavskiy 2019 | √ | √ | √ | √ | X | √ | X | X | √ | √ | 7 |
| Chittleborough 2020 | √ | √ | √ | √ | X | √ | X | √ | X | √ | 7 |
| Dehghani 2019 | √ | √ | √ | √ | √ | √ | √ | X | X | X | 7 |
| Edwards 2010 | √ | √ | √ | √ | √ | √ | X | X | X | √ | 7 |
| Enayatrad 2018 | √ | √ | √ | √ | √ | √ | X | X | X | √ | 7 |
| Exarchakou 2019 | √ | √ | √ | √ | √ | √ | X | X | X | √ | 7 |
| Feletto 2019 | √ | √ | √ | √ | X | √ | X | √ | X | √ | 7 |
| Martinsen 2016 | √ | √ | √ | √ | √ | √ | X | X | X | √ | 7 |
| Giddings 2012 | √ | √ | √ | √ | √ | √ | X | X | X | √ | 7 |
| Loomans-Kropp 2019 | √ | √ | √ | √ | √ | √ | X | X | X | √ | 7 |
| Gandhi 2017 | √ | √ | √ | √ | X | √ | X | √ | X | √ | 7 |
| Lopez 2019 | √ | √ | √ | √ | √ | √ | X | X | X | √ | 7 |
| Lopez-Abente 2010 | √ | √ | √ | √ | √ | √ | X | X | X | √ | 7 |
| McClements 2012 | √ | √ | √ | √ | √ | √ | X | X | X | √ | 7 |
| Ladabaum 2014 | √ | √ | √ | √ | √ | √ | X | X | X | √ | 7 |
| Liu 2015 | √ | √ | √ | √ | √ | √ | √ | X | X | X | 7 |
| Fournel 2016 | √ | √ | √ | √ | X | √ | X | √ | X | √ | 7 |
| Lee 2019 | √ | √ | √ | √ | √ | √ | X | X | X | √ | 7 |
| Khiari; Ben Ayoube 2017 | √ | √ | √ | √ | √ | √ | X | √ | X | X | 7 |
| Jandova 2016 | √ | √ | √ | √ | √ | √ | X | X | X | √ | 7 |
| Li; Lin 2017 | √ | √ | √ | √ | √ | √ | X | X | X | √ | 7 |
| Meza 2010 | √ | √ | √ | √ | √ | √ | X | X | X | √ | 7 |
| Jafri 2013 | √ | √ | √ | √ | √ | √ | X | X | X | √ | 7 |
| Meyer 2010 | √ | √ | √ | √ | X | √ | X | √ | X | √ | 7 |
| Garcia 2018 | √ | √ | √ | √ | X | √ | X | √ | X | √ | 7 |
| Fournel 2012 | √ | √ | √ | √ | X | √ | X | √ | X | √ | 7 |
| Brenner 2019 | √ | √ | √ | √ | √ | √ | X | X | X | √ | 7 |
| Fedewa 2019 | √ | √ | √ | √ | X | √ | X | √ | X | √ | 7 |
| Domati 2014 | √ | √ | √ | √ | X | √ | √ | √ | X | X | 7 |
| Vuik 2019 | √ | √ | √ | √ | √ | √ | X | X | X | √ | 7 |
| Singh 2018 | √ | √ | √ | √ | √ | √ | X | X | X | √ | 7 |
| Nfonsam 2015 | √ | √ | √ | √ | √ | √ | X | X | X | √ | 7 |
| Sammour 2009 | √ | √ | √ | √ | X | √ | √ | X | X | √ | 7 |
| Sheneman 2017 | √ | √ | √ | √ | √ | √ | X | X | X | √ | 7 |
| Murphy 2011 | √ | √ | √ | √ | √ | √ | X | X | X | √ | 7 |
| Siegel; Fedewa 2017 | √ | √ | √ | √ | √ | √ | X | X | X | √ | 7 |
| Siegel 2012 | √ | √ | √ | √ | √ | √ | X | X | X | √ | 7 |
| Sierra 2016 | √ | √ | √ | √ | √ | √ | X | X | X | √ | 7 |
| Oliveira 2016 | √ | √ | √ | √ | √ | √ | X | X | X | √ | 7 |
| Reggiani-Bonetti 2013 | √ | √ | √ | √ | X | √ | √ | √ | X | X | 7 |
| Phipps 2012 | √ | √ | √ | √ | √ | √ | X | X | X | √ | 7 |
| Innos 2018 | √ | √ | √ | √ | √ | √ | X | X | X | √ | 7 |
| Sia 2014 | √ | √ | √ | √ | √ | √ | X | X | X | √ | 7 |
| Stock 2012 | √ | √ | √ | √ | √ | √ | X | X | X | √ | 7 |
| Sun 2020 | √ | √ | √ | √ | √ | √ | X | X | X | √ | 7 |
| Tawadros 2015 | √ | √ | √ | √ | √ | √ | X | X | X | √ | 7 |
| Thirunavukarasu 2010 | √ | √ | √ | √ | √ | √ | X | X | X | √ | 7 |
| Troeung 2017 | √ | √ | √ | √ | √ | √ | X | X | X | √ | 7 |
| Veruttipong 2012 | √ | √ | √ | √ | X | √ | X | √ | X | √ | 7 |
| Wang 2017 | √ | √ | √ | √ | √ | √ | X | X | X | √ | 7 |
| Wang; de Grubb 2017 | √ | √ | √ | √ | √ | √ | X | X | X | √ | 7 |
| Wang 2019 | √ | √ | √ | √ | √ | √ | X | X | X | √ | 7 |
| Wessler 2010 | √ | √ | √ | √ | √ | √ | X | X | X | √ | 7 |
| Yoon 2015 | √ | √ | √ | √ | √ | √ | X | X | X | √ | 7 |
| Zheng 2014 | √ | √ | √ | √ | √ | √ | √ | X | X | X | 7 |
| Zhou 2015 | √ | √ | √ | √ | √ | √ | X | X | X | √ | 7 |
| Zhu 2013 | √ | √ | √ | √ | √ | √ | X | X | X | √ | 7 |
| Zorzi 2019 | √ | √ | √ | √ | √ | √ | X | X | X | √ | 7 |
| Zorzi 2015 | √ | √ | √ | √ | √ | √ | X | X | X | √ | 7 |
| Abdifard 2013 | √ | √ | √ | √ | √ | √ | X | X | X | X | 6 |
| Abreu 2010 | √ | √ | √ | √ | √ | √ | X | X | X | X | 6 |
| Araghi 2018 | √ | √ | √ | √ | X | √ | X | X | X | √ | 6 |
| Ashktorab 2016 | √ | √ | √ | √ | X | √ | X | X | X | √ | 6 |
| Baniasadi 2015 | √ | √ | √ | √ | X | √ | √ | X | X | X | 6 |
| Bhurgri 2011 | √ | √ | √ | √ | √ | √ | X | X | X | X | 6 |
| Winther 2016 | √ | √ | √ | √ | √ | √ | X | X | X | X | 6 |
| Chatterjee 2015 | √ | √ | √ | √ | X | √ | X | X | X | √ | 6 |
| Alsanea 2015 | √ | √ | √ | √ | X | √ | X | X | X | √ | 6 |
| Chauvenet 2011 | √ | √ | √ | √ | √ | √ | X | X | X | X | 6 |
| Chen 2012 | √ | √ | √ | √ | X | √ | X | X | X | √ | 6 |
| Chong 2015 | √ | √ | √ | √ | X | √ | X | X | X | √ | 6 |
| Clarke 2014 | √ | √ | √ | √ | √ | √ | X | X | X | X | 6 |
| Ellis 2018 | √ | √ | √ | √ | X | √ | X | X | X | √ | 6 |
| Eser 2018 | √ | √ | √ | √ | √ | √ | X | X | X | X | 6 |
| May 2017 | √ | √ | √ | √ | X | √ | X | X | X | √ | 6 |
| Koblinski 2018 | √ | √ | √ | √ | X | √ | X | X | X | √ | 6 |
| Missaoui 2011 | √ | √ | √ | √ | √ | √ | X | X | X | X | 6 |
| Kelly 2012 | √ | √ | √ | √ | √ | √ | X | X | X | X | 6 |
| Gan 2019 | √ | √ | √ | √ | X | √ | X | X | X | √ | 6 |
| Fowler 2018 | √ | √ | √ | √ | X | √ | X | X | X | √ | 6 |
| Meester 2019 | √ | √ | √ | √ | X | √ | X | X | X | √ | 6 |
| Li 2017 | √ | √ | √ | √ | X | √ | X | X | X | √ | 6 |
| Khiari 2017 | √ | √ | √ | √ | √ | √ | X | X | X | X | 6 |
| Shadmani 2017 | √ | √ | √ | √ | X | √ | X | X | X | √ | 6 |
| Merrill 2011 | √ | √ | √ | √ | √ | √ | X | X | X | X | 6 |
| McDevitt 2017 | √ | √ | √ | √ | √ | √ | X | X | X | X | 6 |
| Khachfe 2019 | √ | √ | √ | √ | X | √ | X | X | X | √ | 6 |
| Brenner 2017 | √ | √ | √ | √ | √ | √ | X | X | X | X | 6 |
| Augustus 2018 | √ | √ | √ | √ | X | √ | X | X | X | √ | 6 |
| Davis 2011 | √ | √ | √ | √ | X | √ | X | X | X | √ | 6 |
| Siegel 2017 | √ | √ | √ | √ | √ | √ | X | X | X | X | 6 |
| Savijarvi 2019 | √ | √ | √ | √ | X | √ | X | X | X | √ | 6 |
| Van Beck 2018 | √ | √ | √ | √ | X | √ | X | X | X | √ | 6 |
| Russo 2019 | √ | √ | √ | √ | √ | √ | X | X | X | X | 6 |
| Oliphant 2011 | √ | √ | √ | √ | X | √ | X | X | X | √ | 6 |
| Perdue 2014 | √ | √ | √ | √ | X | √ | X | X | X | √ | 6 |
| Murphy 2017 | √ | √ | √ | √ | X | √ | X | X | X | √ | 6 |
| Siegel 2020 | √ | √ | √ | √ | √ | √ | X | X | X | X | 6 |
| Shin 2012 | √ | √ | √ | √ | √ | √ | X | X | X | X | 6 |
| Murphy 2018 | √ | √ | √ | √ | X | √ | X | X | X | √ | 6 |
| Sung 2019 | √ | √ | √ | √ | X | √ | X | X | X | √ | 6 |
| Rafiemanesh 2016 | √ | √ | √ | √ | √ | √ | X | X | X | X | 6 |
| Nowicki 2018 | √ | √ | √ | √ | √ | √ | X | X | X | X | 6 |
| Oppelt 2019 | √ | √ | √ | √ | X | √ | X | X | X | √ | 6 |
| Murphy 2019 | √ | √ | √ | √ | X | √ | X | X | X | √ | 6 |
| Siegel 2014 | √ | √ | √ | √ | √ | √ | X | X | X | X | 6 |
| Rejali 2018 | √ | √ | √ | √ | √ | √ | X | X | X | X | 6 |
| Sarakarn 2017 | √ | √ | √ | √ | √ | √ | X | X | X | X | 6 |
| Keum 2014 | √ | √ | √ | √ | X | √ | X | X | X | √ | 6 |
| Singh 2014 | √ | √ | √ | √ | X | √ | X | X | X | √ | 6 |
| Sjostrom 2018 | √ | √ | √ | √ | X | √ | X | X | X | √ | 6 |
| Stromberg 2019 | √ | √ | √ | √ | X | √ | X | X | X | √ | 6 |
| Thuraisingam 2017 | √ | √ | √ | √ | X | √ | X | X | X | √ | 6 |
| Ugarte 2012 | √ | √ | √ | √ | √ | √ | X | X | X | X | 6 |
| Ullah 2018 | √ | √ | √ | √ | X | √ | X | X | X | √ | 6 |
| Wan Ibrahim 2020 | √ | √ | √ | √ | X | √ | X | X | X | √ | 6 |
| Wen 2018 | √ | √ | √ | √ | √ | √ | X | X | X | X | 6 |
| Yee 2010 | √ | √ | √ | √ | √ | √ | X | X | X | X | 6 |
| Yeo 2017 | √ | √ | √ | √ | X | √ | X | X | X | √ | 6 |
| Zhang 2018 | √ | √ | √ | √ | X | √ | X | X | X | √ | 6 |
| Ohri 2020 | √ | √ | √ | √ | X | √ | X | X | X | √ | 6 |
| Hassan 2016 | √ | √ | √ | √ | x | √ | X | X | X | X | 5 |
| Al Dahhan 2018 | √ | √ | √ | √ | X | √ | X | X | X | X | 5 |
| Bailey 2015 | √ | √ | √ | √ | X | √ | X | X | X | X | 5 |
| Crocetti 2010 | √ | √ | √ | √ | X | √ | X | X | X | X | 5 |
| Hasanpour-Heidari 2019 | √ | √ | √ | √ | X | √ | X | X | X | X | 5 |
| Lemmens 2010 | √ | √ | √ | √ | X | √ | X | X | X | X | 5 |
| Fusco 2010 | √ | √ | √ | √ | X | √ | X | X | X | X | 5 |
| Klugarova 2019 | √ | √ | √ | √ | X | √ | X | X | X | X | 5 |
| Klimczak 2011 | √ | √ | √ | √ | X | √ | X | X | X | X | 5 |
| Koblinski 2019 | √ | √ | √ | √ | X | √ | X | X | X | X | 5 |
| Purim 2013 | √ | √ | √ | √ | X | √ | X | X | X | X | 5 |
| Rahman 2015 | √ | √ | √ | √ | X | √ | X | X | X | X | 5 |
| Mosli 2012 | √ | √ | √ | √ | X | √ | X | X | X | X | 5 |
| Mosli 2012 | √ | √ | √ | √ | X | √ | X | X | X | X | 5 |
| Pakzad 2016 | √ | √ | √ | √ | X | √ | X | X | X | X | 5 |
| Pescatore 2013 | √ | √ | √ | √ | X | √ | X | X | X | X | 5 |
| Palmieri 2013 | √ | √ | √ | √ | X | √ | X | X | X | X | 5 |
| Paquette 2015 | √ | √ | √ | √ | X | √ | X | X | X | X | 5 |
| Zhabagin 2015 | √ | √ | √ | √ | X | √ | X | X | X | X | 5 |
