# Supplementary material for: A systematic review of methods to estimate colorectal cancer incidence using population-based cancer registries
Source: BMC Med Res Methodol. 2022 May 19;22:144. doi: 10.1186/s12874-022-01632-7 (PMC9118801; doi:10.1186/s12874-022-01632-7)
Supplement: Supplementary file 5 — Additional file 5. Search strategy. [file 12874_2022_1632_MOESM5_ESM.docx]

**Additional file 5** Search strategy (P.1-3)

**Database: MEDLINE**

In steps:

| **#** | **Exposures** | **Hits** |
| --- | --- | --- |
| 1 | exp Colorectal Neoplasms/ | 199011 |
| 2 | (colorect* or rect* or colon* or bowel).tw,kw. | 821785 |
| 3 | (cancer* or neoplas* or tumo?r* or malignan* or carcinoma* or adeno*).tw,kw. | 3493361 |
| 4 | 2 and 3 | 329291 |
| 5 | 1 or 4 | 369542 |
| 6 | Incidence/ | 258624 |
| 7 | incidence.tw,kw. | 740036 |
| 8 | Trend*.tw,kw. | 391905 |
| 9 | 6 or 7 or 8 | 844856 |
| 10 | exp Registries/ | 94470 |
| 11 | (cancer adj3 regist*).tw,kw. | 23493 |
| 12 | 10 or 11 | 107181 |
| 13 | 5 and 9 and 12 | 3318 |
| 14 | limit 13 to english language | 3130 |
| 15 | limit 14 to yr="2010 -Current" | **1787** |

**Database: Embase**

In steps:

| **#** | **Exposures** | **Hits** |
| --- | --- | --- |
| 1 | (colorect* or rect* or colon* or bowel).tw,kw. | 925983 |
| 2 | (cancer* or neoplas* or tumo?r* or malignan* or carcinoma* or adeno*).tw,kw. | 3825267 |
| 3 | 1 and 2 | 429033 |
| 4 | exp colon tumor/ | 280525 |
| 5 | exp rectum tumor/ | 229465 |
| 6 | 3 or 4 or 5 | 494055 |
| 7 | Incidence/ | 376884 |
| 8 | Cancer incidence/ | 67144 |
| 9 | incidence.tw,kw. | 885965 |
| 10 | Trend*.tw,kw. | 502568 |
| 11 | 7 or 8 or 9 or 10 | 1460125 |
| 12 | Cancer registry/ | 33889 |
| 13 | Register/ | 108956 |
| 14 | (cancer adj3 regist*).tw,kw. | 33651 |
| 15 | 12 or 13 or 14 | 150261 |
| 16 | 6 and 11 and 15 | 4657 |
| 17 | limit 16 to english language | 4444 |
| 18 | Limit 17 to conference abstract | 1440 |
| 19 | 17 NOT 18 | 3004 |
| 20 | limit 19 to yr="2010 -Current" | **1977** |

**Database: Web of science**

In steps:

| **#** | **Exposures** | **Hits** |
| --- | --- | --- |
| 1 | (TS=(cancer* or neoplas* or tumor* or tumours or malignan* or carcinoma* or adeno*)) AND LANGUAGE: (English)  Indexes=SCI-EXPANDED, SSCI, A&HCI, CPCI-S, CPCI-SSH, ESCI, CCR-EXPANDED, IC Timespan=2010-2020 | 2,126,653 |
| 2 | (TS=(colorect* or rect* or colon* or bowel)) AND LANGUAGE: (English)  Indexes=SCI-EXPANDED, SSCI, A&HCI, CPCI-S, CPCI-SSH, ESCI, CCR-EXPANDED, IC Timespan=2010-2020 | 667,363 |
| 3 | 1 and 2 | 235,826 |
| 4 | (TS=(incidence or trend*)) AND LANGUAGE: (English)  Indexes=SCI-EXPANDED, SSCI, A&HCI, CPCI-S, CPCI-SSH, ESCI, CCR-EXPANDED, IC Timespan=2010-2020 | 883,278 |
| 5 | (TS=(registry or registries)) AND LANGUAGE: (English)  Indexes=SCI-EXPANDED, SSCI, A&HCI, CPCI-S, CPCI-SSH, ESCI, CCR-EXPANDED, IC Timespan=2010-2020 | 104,506 |
| 6 | 3 and 4 and 5 | **1584** |
